# Alchemical Free Energy Calculations to Investigate Protein–Protein Interactions: the Case of the CDC42/PAK1 Complex

**DOI:** 10.1021/acs.jcim.2c00348

**Published:** 2022-06-09

**Authors:** Maria Antonietta La Serra, Pietro Vidossich, Isabella Acquistapace, Anand K. Ganesan, Marco De Vivo

**Affiliations:** † Laboratory of Molecular Modeling and Drug Discovery, Istituto Italiano di Tecnologia, via Morego 30, Genoa 16163, Italy; ‡ Department of Dermatology, University of California, Irvine, Irvine, California 92697, United States; § Department of Biological Chemistry, University of California, Irvine, Irvine, California 92697, United States

## Abstract

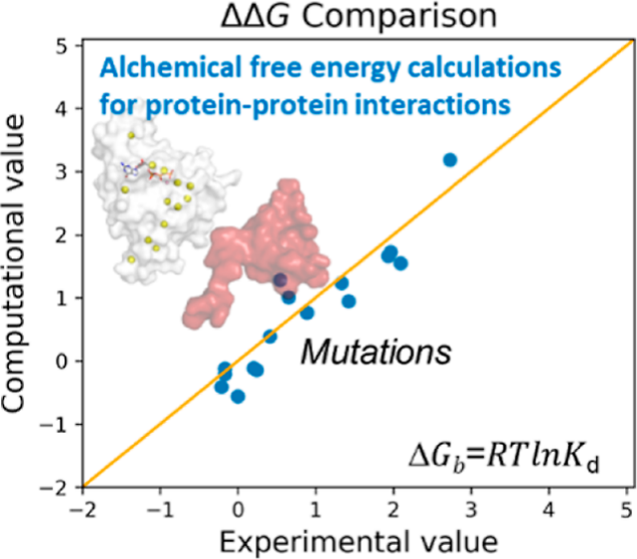

Here, we show that alchemical free energy calculations can quantitatively compute the effect of mutations at the protein–protein interface. As a test case, we have used the protein complex formed by the small Rho-GTPase CDC42 and its downstream effector PAK1, a serine/threonine kinase. Notably, the CDC42/PAK1 complex offers a wealth of structural, mutagenesis, and binding affinity data because of its central role in cellular signaling and cancer progression. In this context, we have considered 16 mutations in the CDC42/PAK1 complex and obtained excellent agreement between computed and experimental data on binding affinity. Importantly, we also show that a careful analysis of the side-chain conformations in the mutated amino acids can considerably improve the computed estimates, solving issues related to sampling limitations. Overall, this study demonstrates that alchemical free energy calculations can conveniently be integrated into the design of experimental mutagenesis studies.

## Introduction

Protein–protein interactions are involved in key biological functions, including cell regulation and signaling.^1,2^ Such non-covalent associations between protein partners are dynamic and specific events through which cells receive, integrate, and distribute regulatory information. The affinity between the two proteins results from the particular shape and physico-chemical complementarity between the two protein interfaces in contact,^2^ in addition to other environmental factors.^3,4^ Thus, investigating protein–protein interactions is of significant interest in biochemistry and drug discovery.^5−7^

Mutagenesis is undoubtedly a leading experimental technique for the study of protein–protein interactions. Such experiments can reveal key protein–protein interactions that, upon their mutations, affect protein–protein binding the most. However, the number of possible mutants to consider can be excessively high. In this regard, computational methods capable of predicting the effects of mutations and quantifying the binding affinity between proteins would help rank the most relevant mutations to validate in experimental studies. Structural bioinformatics tools to address this problem have indeed been reported over the years.^2,8,9^ On the other hand, given the great improvements in computing performance, atomistic molecular dynamics (MD) simulations are also a suitable computational approach for such predictions, thus allowing us to take protein flexibility fully into account. For example, classical MD or Monte Carlo simulations can sample the system’s configurational space and predict the protein–protein binding free energy change upon mutation of specific residues at the interface. These simulation methods can be used to run alchemical free energy calculations, which exploit “unphysical” transformations between end-states.^10−13^ Notably, such an alchemical approach is routinely and successfully used to design small-molecule drugs.^13−18^ Its use to rationalize or predict the effect of protein mutations on drug or substrate binding is also well established, dating back to early applications of the method in biochemistry.^19^ Despite this, its use in the context of protein–protein interactions is limited. Only recently, Friesner and co-workers have reported a study exploring the performance of alchemical free energy calculations for investigating mutations at antibody/glycoprotein interfaces, with encouraging results for the design of tailored antibodies.^20,21^ Interestingly, the application of physics-based free energy methods has been described in the context of protein–peptide binding.^22,23^

In this study, we have used alchemical free energy calculations to investigate the interface between two signaling proteins, namely CDC42 and PAK1. Such protein interactions are involved in fundamental cellular processes such as proliferation, mobility, and survival.^24^ Also, disruption of the CDC42/PAK1 complex is a promising strategy for cancer drug discovery.^25^ Specifically, deregulation of PAK1 due to its hyperactivation has been reported in cancer cells and is associated with cancer development and carcinogenesis.^26^ This hyperactivation is often caused by the upregulation and/or the overexpression of CDC42, making the CDC42/PAK1 interaction a favorable target to treat cancer. For these reasons, such a complex has been extensively characterized experimentally over the last couple of decades, generating a wealth of structural, mutagenesis, and binding affinity data that elect the CDC42/PAK1 complex as a valuable test case for alchemical free energy calculations to investigate the effect of mutations on protein–protein interaction.^27^

In detail, CDC42 is a GTPase of the small G protein family. CDC42 acts as a signaling protein, interconverting between inactive (GDP-bound) and active (GTP-bound) states.^28^ Structurally, small G proteins like CDC42 share a central core with five α-helices and six β-strands linked by loops. Five conserved motifs (GxxxxGKS/T; T; DxxG; N/TKx; SAK) stabilize the nucleotide in the binding site (Figure S1A). The active and inactive states differ in the so-called switch region, which assumes the proper structural arrangement for binding downstream effector proteins, such as kinases, only in the GTP-bound state.^29−31^ Indeed, PAK1 is a serine/threonine kinase, which regulates the activity of other proteins through their phosphorylation.^32^ Structurally, PAK1 contains a highly conserved C-terminal catalytic kinase domain and an N-terminal regulatory domain. The latter includes a conserved CRIB domain (CDC42/RAC interacting binding, *I–S–X–P–(X)*_*2–4*_*–F–X–H–X–X–H–V–G*) and an auto-inhibitory domain (AID). The inactive dimeric conformation of PAK1 is trans-inhibited (Figure S1B), with the AID of a monomer binding to the catalytic domain of the other monomer of PAK1 and *vice versa*. This dimeric form is disrupted by the binding of GTP-bound active CDC42/RAC to the CRIB domain, followed by the auto-phosphorylation of a threonine residue (Thr423).^24,33,34^ This process leads to the activated state of PAK1.

Here, we report a benchmark study of alchemical free energy calculations used to estimate the change in affinity for 16 reported mutations of CDC42, which was used to investigate their association with PAK1. Comparison with available experimental data^27^ shows that this computational approach can be routinely used to design and prioritize mutagenesis experiments and investigate protein–protein interactions in signaling networks.

## Methods

### Model Systems

**Figure 1:**
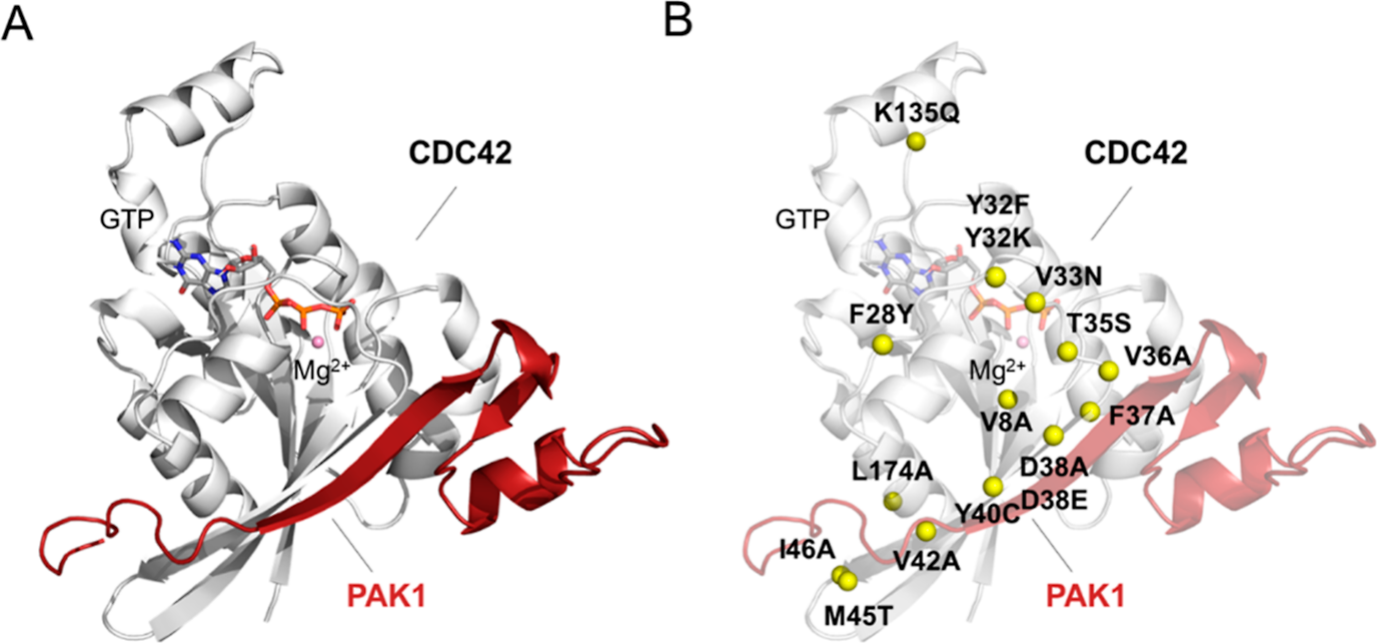
Structural representation of the CDC42/PAK1 model. (A) CDC42/PAK1 complex is reported (see Methods section). CDC42 is represented as a white cartoon, while PAK1 is shown in red. The GTP nucleotide and the Mg^2+^ ion are in sticks and balls, respectively. (B) Analyzed single-point mutations are represented as yellow balls on the CDC42/PAK1 complex structure.

The experimental structure of CDC42 in the complex with the CRIB domain of PAK6 (PDB code 2ODB, 2.4 Å resolution) was used as a template for comparative modeling to build a model of the CDC42/PAK1 complex (Figure 1A), as well as to set up a model of CDC42 alone by removing the effector. For CDC42, residues 2 to 178 were considered, excluding the flexible carboxyl-terminal region, which regulates homodimer formation and the proper subcellular localization but is not involved in the binding of effectors.^35,36^ For PAK1, modeling included residues 70 to 117, which have been proven to comprise the smallest PAK1 fragment required for the interaction with CDC42.^37^ Comparative modeling was performed with MODELLER version 10.1.^38^ The model with the lowest DOPE score was selected for system setup. To assess the reliability of the model, we carried out a structural analysis of available X-ray structures of GTPases/PAK complexes^39^ (Figure S2A). As quantified by the root mean square deviation (RMSD) of interfacial residues, no major structural variations were observed between the model and experimental structures (Figure S2B). Furthermore, conserved contacts established between the interface β-sheets of CDC42 and PAK were maintained in the model (Figure S2C), highlighting the consistency of our structural model with known structures of homologous complexes. The GTP substrate, catalytic Mg^2+^ ion, and experimentally determined water molecules at the active site were included in both the apo and PAK1-bound CDC42 forms. Systems were solvated in cubic simulation boxes extending at least 14 Å from the protein surface. Sodium ions were added randomly to neutralize the charge of the systems. Models of CDC42 Y40C and F37A variants in a PAK1-bound form were built from the *wild-type* (*wt*) model. Final models included ∼60,000 atoms in a 85 × 85 × 85 Å^3^ box for apo CDC42 and ∼78,500 atoms in a ∼93 × 93 × 93 Å^3^ box for the complexes.

### MD Simulations

MD simulations were performed with the pmemd module of AMBER20.^40^ The AMBER-ff14SB force field^41^ was used for the protein, while parameters from recent literature were adopted for GTP and Mg^2+^.^42,43^ Monovalent ions were described with Joung–Cheatham parameters,^44^ and the TIP3P model^45^ was used for water. Simulations were performed with a distance cutoff of 10 Å. Long-range electrostatics were treated with the particle mesh Ewald method. Bonds involving hydrogen atoms were constrained, allowing a time step of 2 fs. After solvent equilibration, systems were energy minimized and gently heated to 303 K for 0.5 ns while restraining protein backbone atoms to stay close to the experimental structure. The Andersen-like temperature-coupling scheme^46^ and a Monte Carlo barostat were used to maintain temperature and pressure close to room temperature conditions. About 1 μs of MD simulations in the *NPT* ensemble were accumulated for each system.

### Alchemical Free Energy Calculations

Binding free energies (Δ*G*_b_) between mutated forms of CDC42 and the binding domain of PAK1 were computed with respect to the *wt* enzyme [relative binding free energies ΔΔ*G*_b_ = Δ*G*_b_ (mutated CDC42) – Δ*G*_b_ (*wt* CDC42)] using alchemical transformations.^47^ Accordingly, CDC42 was transformed from *wt* into the mutant in both the apo and PAK1-bound forms. The free energy change associated with each transformation was estimated using thermodynamic integration (eq 1),^48^ and their difference provided an estimate of ΔΔ*G*_b_ (see thermodynamic cycle in Figure S3A).
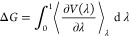
116 reported mutations were considered^27^ (Table 1 and Figure 1B). Binding free energies (Δ*G*_b_) were computed from experimental data using the measured *K*_d_ value and the equation Δ*G*_b_ = *RT* ln *K*_d_. Experimental ΔΔ*G*_b_ values were then obtained by using the calculated Δ*G*_b_ for the *wt* and mutated forms of CDC42 in the CDC42/PAK1 complex.

**Table 1:** Equilibrium Constants^27^ and Relative Binding Free Energy Values for the Association of CDC42 Mutants and PAK1^a^

mutation	*K*_d_	ΔΔ*G*_b_^exp^	ΔΔ*G*_b_^comp^	ΔΔ*G*_b_^MM/GBSA^
wild-type	20 ± 4	0 ± 0.12	–0.56 ± 0.16	
V8A	14 ± 4	–0.21 ± 0.17	–0.41 ± 0.04	0.01 ± 0.02**
F28Y	15 ± 5	–0.17 ± 0.20	–0.20 ± 0.05	
Y32F	90 ± 50	0.89 ± 0.33	0.88 ± 0.12	
Y32K	680 ± 90	2.09 ± 0.08	1.55 ± 0.22	
V33N	28 ± 5	0.20 ± 0.11	–0.11 ± 0.12	
T35S	520 ± 82	1.93 ± 0.09	1.67 ± 0.05	
V36A	220 ± 13	1.42 ± 0.03	0.95 ± 0.07	2.82 ± 0.65**
F37A	190 ± 23	1.33 ± 0.07	1.24 ± 0.16	–0.27 ± 0.12
				4.11 ± 1.03**
D38A	>2000	2.73*	3.19 ± 0.23	14.28 ± 1.54**
D38E	550 ± 53	1.96 ± 0.06	1.73 ± 0.17	
Y40C	>1000	2.32*	3.94 ± 0.19	–0.95 ± 0.15
V42A	40 ± 8	0.41 ± 0.12	0.39 ± 0.08	2.26 ± 0.74**
M45T	30 ± 4	0.24 ± 0.08	–0.14 ± 0.09	
I46A	60 ± 8	0.65 ± 0.08	1.01 ± 0.11	1.53 ± 0.58**
K135Q	15 ± 5	–0.17 ± 0.2	–0.12 ± 0.20	
L174A	250 ± 9	0.54 ± 0.11	1.28 ± 0.12	0.69 ± 0.43 **

a The examined single-point mutations are reported together with their *K*_d_ equilibrium constants, experimental ΔΔ*G*_b_ values, and the computed ΔΔ*G*_b_ values through alchemical free energy calculations and MM/GBSA methods. * symbol indicates the absence of an estimated experimental error. ** symbol indicates that the value has been calculated by alanine scanning.

Alchemical calculations were started from equilibrated configurations (see Results section) from equilibrium MD simulations of the CDC42/PAK1 complex and CDC42 alone. Each transformation was carried out in 12 windows (corresponding to λ values: 0.00922, 0.04794, 0.11505, 0.20634, 0.31608, 0.43738, 0.56262, 0.68392, 0.79366, 0.88495, 0.95206, and 0.99078 and weights 0.02359, 0.05347, 0.08004, 0.10158, 0.11675, 0.12457) performing 10 ns simulations at each λ value. Bonds were not constrained, requiring an integration time step of 1 fs. Backbone atoms of the residues involved in the mutations were transformed linearly, while side-chain atoms were treated with softcore potentials^49^ for both Lennard-Jones and electrostatic interactions. For certain mutations, different atom mapping schemes were considered (see Results section). Some mutations involve a change of charge in the system. To treat these cases, we adopted the alchemical co-ion approach:^50,51^ when a negative charge was annealed (D38A), concomitantly a Na^+^ ion was converted into a water molecule; when a positive charge was annealed (K135Q), concomitantly a water molecule was converted into a Na^+^ ion; and when a positive charge was created (Y32K), concomitantly a Na^+^ ion was converted into a water molecule (see Figure S3B). Transformations were performed at constant volume (the equilibrated volume from MD simulations) and temperature. Data analysis was performed after discarding the first 10% of the simulation time (corresponding to the first ns of simulation) of each window. In order to estimate errors on Δ*G* (eq 1) the time series of ∂*V*/∂λ, values from each window were re-sampled to obtain uncorrelated samples,^52^ from which averages and variances were computed. The error on ΔΔ*G*_b_ was obtained by combining the errors of the individual transformations. The convergence of the computed ΔΔ*G*_b_ was assessed by estimating it as a function of simulation time, considering intervals both in the forward and the reverse direction^53^ (Figure S10).

### MM/GBSA Calculations

Implicit solvent calculations (generalized Born model in the Onufriev–Bashford–Case formulation)^54,55^ were combined with vacuum molecular mechanical energy evaluations to estimate Δ*G*_b_. Calculations were performed for the CDC42/PAK1 complex and for each partner separately using configurations from the equilibrium MD simulations of the *wt* CDC42/PAK1 complex and CDC42 Y40C and F37A variants. The *wt* CDC42/PAK1 trajectory was also used to evaluate the effect of mutating certain residues into alanine (alanine scanning^56^). All steps required by the calculation were automatized with MM/PBSA.py distributed with AmberTools.^57^ A 0 M ion concentration was used in the GB calculation, and the linear combination of a pairwise overlap method^58^ was used to calculate the molecular surface area.

## Results

### Equilibrium MD Simulations of CDC42 in Complex with PAK1

We analyzed the stability and main structural interactions of the CDC42/PAK1 complex using equilibrium MD simulations. First, we considered the *wt* of the CDC42/PAK1 complex for a total running time of 1 μs. The complex equilibrated after ∼15 ns and remained stable for the rest of the simulation (Figure S4A). The initial GTP binding pose and the coordination of the catalytic Mg^2+^ ion were also well maintained throughout the simulations (Figure S4B). In particular, the CDC42/PAK1 protein interface appeared highly stable compared to the unbound CDC42, which exhibited larger fluctuations of the residues 35–72 belonging to the switch motifs and to the β-sheet in contact with PAK1 (Figure S4C).

Additionally, to explore the impact of single-point mutations on the stability of the complex, we ran further equilibrium simulations of the CDC42/PAK1 complex with either the Y40C or the F37A mutation in CDC42. Both mutations were detrimental to binding.^27^ In particular, the Y40C mutation is among the most harmful mutations, causing a >100-fold increase in *K*_d_. Specifically, the Y40C mutation of CDC42 was observed experimentally to destabilize its binding to PAK1 by >2.3 kcal/mol (calculated by first converting in kcal/mol the experimental *K*_d_ value of >1000 nM—see Methods section). The F37A mutation in CDC42 generated a decrease of 1.3 kcal/mol in the affinity for PAK1 (calculated from the experimental *K*_d_ value of ∼190 nM). Also, F37A exemplifies a mutation often explored in mutagenesis studies, in which the bulky residue Phe is changed to the smaller apolar Ala residue.^59^

**Figure 2:**
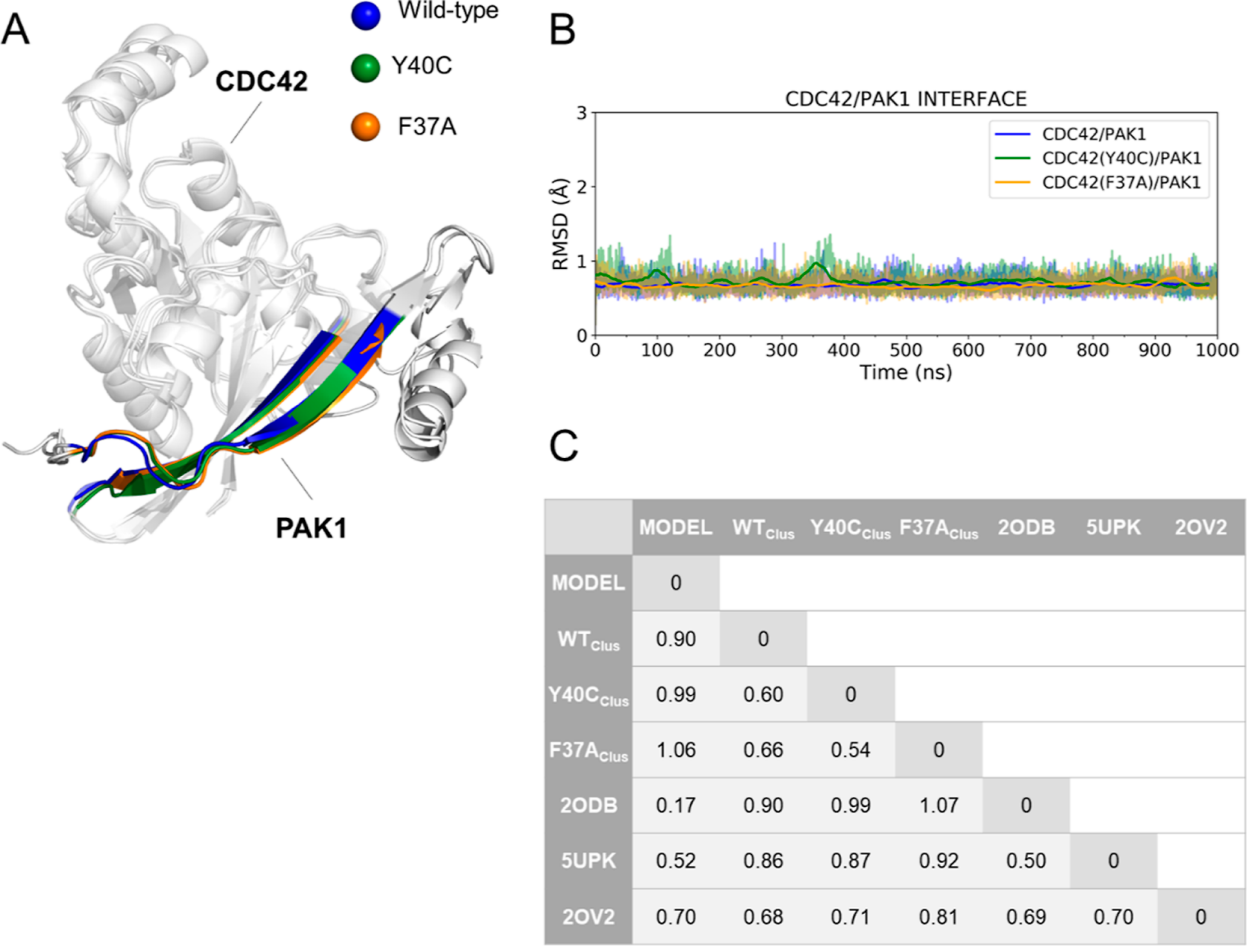
RMSD analysis of the interface of the CDC42/PAK1 systems. (A) Structural alignment of representative conformations from MD simulations. The protein structures are represented as a white cartoon, while the GTP nucleotide and Mg^2+^ ion are illustrated as sticks and balls, respectively. Interface residues on both CDC42 and PAK1 are highlighted as blue, green, and orange for the *wt*, Y40C, and F37A systems, respectively. (B) Time-series RMSD descriptors for CDC42 *wt* (blue), Y40C (green), and F37A (orange) variants are reported. (C) RMSD (in Å) of interface residues between different structures, including MD representative structures, the initial model, and experimental structures.

Structures from all our simulations (*wt* and two mutated systems) were clustered^60^ based on the RMSD of the interface residues, revealing very similar conformations (Figure 2). The switch motifs (switch I and II) of CDC42 maintained the active conformation along the entire simulations, remaining aligned to the initial structure (Figure S5A) in all cases. Also, PAK1 showed no difference in the *wt versus* mutated complexes (Figure S5B) during the simulations. In particular, the β-sheets forming the intermolecular interactions between CDC42 and PAK1 were quite stable. These overall analyses of the MD trajectories were propaedeutic to the alchemical free energy calculations (see next paragraphs).

### Free Energy (ΔΔ*G*_b_) Estimates Based on Side-chain Alchemical Transformations

Having tested our model system and its overall stability in classical MD, we moved to the calculation of the relative binding free energies (ΔΔ*G*_b_) to study the effect of 16 point mutations of CDC42 for which experimental data have been reported^27^ (Table 1). These calculations use the alchemical transformation of one residue in the CDC42 protein alone and in the CDC42/PAK1 complex. Thus, from equilibrated configurations of the CDC42/PAK1 complex and CDC42, a total of 34 systems were built (including a control calculation on the *wt* system) and used to run alchemical transformations carried out in 12 λ-windows of 10 ns each for a total of 120 ns per transformation. In total, a cumulative time of ∼4 μs was collected.

**Figure 3:**
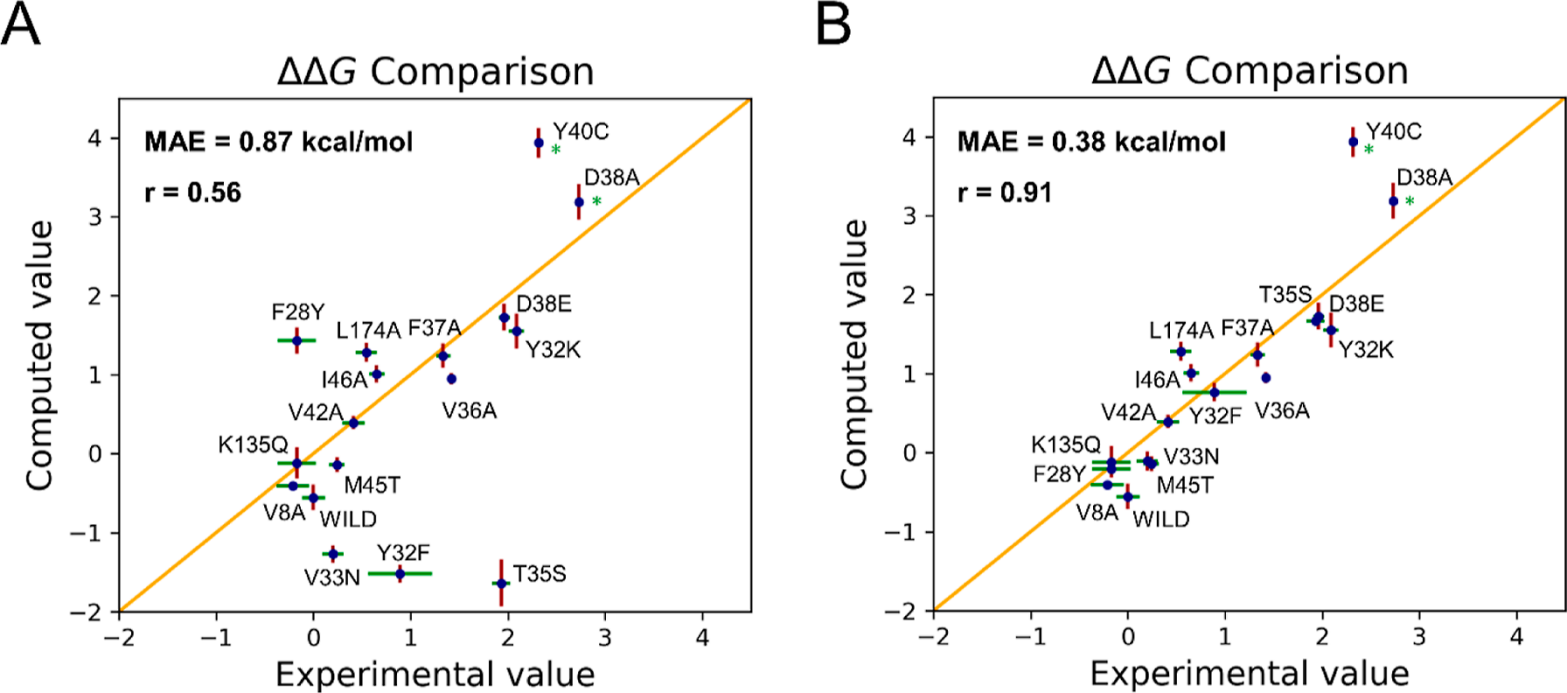
(A) Initial ΔΔ*G*_b_ (in kcal/mol) computed using the alchemical transformations and plotted against the experimental values. (B) Scatter plot obtained after improving the ΔΔ*G*_b_ estimates for T35S, F28Y, Y32F, and V33N (see text for details). In both A and B, the examined single-point mutations are reported together with their computed (red) and experimental (green) error bars. The asterisk (*) marks mutations for which the experimental error was not reported.

All systems remained stable during the alchemical transformations, with low RMSD values for residues at the CDC42/PAK1 interface as well as for the GTP binding pose (Figures S6 and S7). Importantly, the plot in Figure 3A demonstrates a good agreement of the computed ΔΔ*G*_b_ values with experimental data. The mean absolute error (MAE) is 0.87 kcal/mol, which is in the range of successful applications of alchemical free energy calculations in drug design.^13,61,62^

Notably, for 10 mutations out of 16, the error is below 0.5 kcal/mol (Table 1). We found that the CDC42 mutations V8A, V42A, M45T, and K135Q have a marginal effect on the free energy of binding to PAK1. Also, mutations Y32K, D38A, D38E, and Y40C are suggested to disfavor the binding between the two partners, with D38A being particularly detrimental—which is in line with the experimental results (*K*_d_ value > 2000 nM). Overall, our results indicate that alchemical binding free energy calculations can locate those mutations that can affect binding affinity. For example, we note that D38A leads to a >1000-fold lower affinity for PAK1 compared to the *wt* enzyme. Furthermore, the positive sign (indicative of a harmful mutation) of ΔΔ*G*_b_ was predicted correctly for all mutations having a reported ΔΔ*G*_b_ > 0.5 kcal/mol.

Also, the ΔΔ*G*_b_ for CDC42 mutants Y40C and F37A estimated from the alchemical transformations is in good agreement with the experimental data. We computed a value of 3.94 ± 0.04 kcal/mol for Y40C and 1.24 ± 0.30 kcal/mol for F37A (*vs* >2.32 and 1.33 ± 0.07 kcal/mol from experiments, respectively). It is worth noting that initially, we applied MM/GBSA calculations to quantify the Δ*G*_b_ of the CDC42/PAK1 complexation in the *wt*, compared to the mutated systems (using ∼10,000 snapshots from equilibrium MD runs). In this case, the relative binding free energy (ΔΔ*G*_b_) estimates were of −0.27 ± 0.12 and −0.95 ± 0.15 kcal/mol for F37A and Y40C, respectively (Table 1). These estimates match poorly with the Δ*G*_b_ from experiments for such two mutations (see above), indicating the inherent difficulties in quantifying exactly the effect of point mutations at the protein interfase using MM/GBSA. Furthermore, for the mutations to alanine, the comparison between the alchemical transformation results (MAE value of 0.3 kcal/mol) and the computationally cheaper alanine scanning approach (MAE value of 2.7 kcal/mol) demonstrates the better accuracy of alchemical transformations to predict the effect of single-point mutations to alanine in this system.

### Improved ΔΔ*G*_b_ Estimates of Single Point Mutations

Despite the encouraging agreement of the computed free energy changes compared to the experimental data, the estimates for four mutations exhibit deviations larger than the mean error. Namely, this is the case for T35S, F28Y, Y32F, and V33N. This is particularly worrying given that the predicted change is sometimes in the opposite direction with respect to the experimental determination. To address this apparent issue, we started exploring different atom mapping schemes to preserve the key interactions established by the residues involved in single-point mutation.

#### Tuning the Mapping Scheme for T35S and F28Y

**Figure 4:**
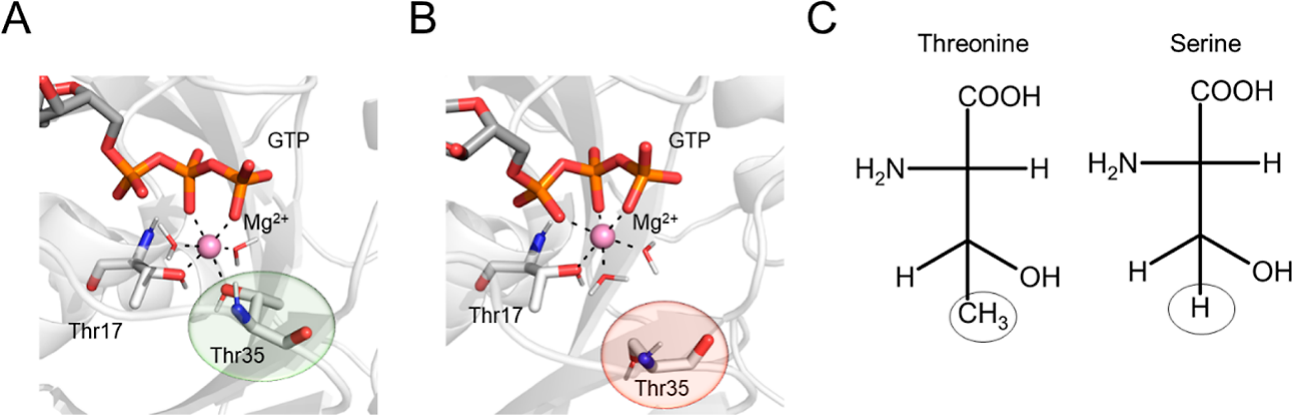
T35S CDC42 variant. (A) Interaction between the catalytic Mg^2+^ and the hydroxyl group of Thr35 as observed in the equilibrium MD simulations of the *wt* system; (B) after the initial alchemical transformation of Thr25 into Ser, the coordination sphere of Mg^2+^ was disrupted. The protein is represented as a white cartoon, the GTP nucleotide and the residues coordinating Mg^2+^ as sticks, and the Mg^2+^ ion as a ball. (C) Revised atom mapping used to improve the ΔΔ*G*_b_ estimate retrieved from alchemical transformation. In this case, the circled atoms are those considered unique for the transformation.

We first report the results on the tuning of the mapping scheme for the T35S mutation. Side-chain alchemical transformations predict this mutation to be favorable by −1.6 ± 0.2 kcal/mol contrary to the experimental determination (1.9 ± 0.1 kcal/mol). The side chain of T35 is bound to the catalytic Mg^2+^, whose coordination sphere is considered fundamental for preserving the active conformation^28,31,63^ (Figure 4A).

During the alchemical transformation of the whole side chain of T35 into a serine residue, the hydroxyl group of the latter does not maintain the initial interaction of T35 with Mg^2+^ (Figure 4B). This leads to a destabilization of the active site. Notably, this occurs in both the bound and unbound CDC42 alchemical transformation calculations (Figure S8A). We thus decided to use a different mapping scheme to transform a threonine into a serine, in which only the terminal methyl group of the threonine and the corresponding hydrogen atom of serine were considered unique to each residue (*i.e.*, the atoms of the −C_β_HOH group were considered common atoms, Figure 4C). With this mapping scheme, the integrity of the Mg^2+^ coordination sphere was maintained during the alchemical transformation (Figure S8B), resulting in a much better ΔΔ*G*_b_ estimate of 1.7 ± 0.1 (*vs* 1.9 ± 0.1 kcal/mol from experiments).

**Figure 5:**
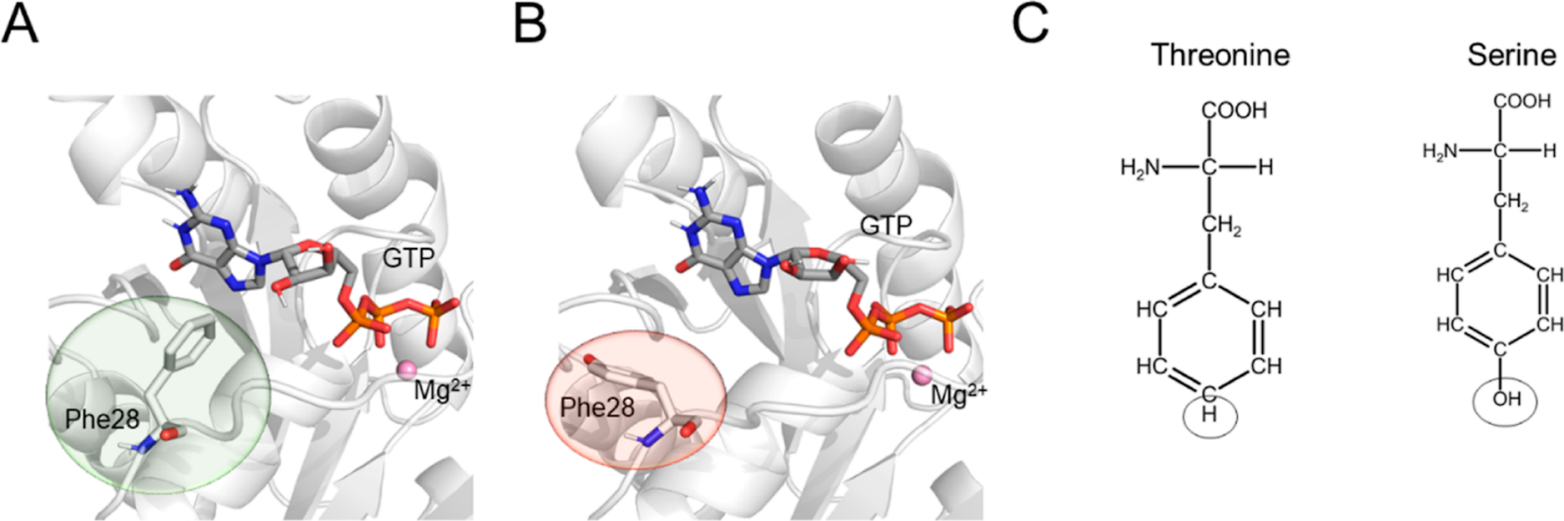
F28Y CDC42 variant. (A) Interaction between GTP and the aromatic ring of Phe28 as observed in the equilibrium MD simulations of the *wt* system; (B) after the initial alchemical transformation of Phe28 into Tyr, the aromatic ring was no longer in contact with GTP. The protein is represented as a white cartoon, the GTP nucleotide and the residues involved in the single-point mutation as sticks, and the Mg^2+^ ion as a ball. (C) Revised atom mapping used to improve the ΔΔ*G*_b_ estimate retrieved from the alchemical transformation. In this case, the circled atoms are those considered unique for the transformation.

A second case is the F28Y mutation. According to the side-chain alchemical transformations, the ΔΔ*G*_b_ for this mutation is disfavored by 1.4 ± 0.2 kcal/mol, differing from the experimental outcome, which shows this single-point mutation to be neutral (−0.2 ± 0.2 kcal/mol). The side chain of the highly conserved F28 is recognized to stabilize the binding of the guanine ring of substrate GTP at the catalytic pocket^63,64^ (Figure 5A).

The analysis of the alchemical transformation trajectories revealed larger fluctuations of the sidechain rings compared to the equilibrium MD simulations of the *wt* enzyme (Figures 5B and S9A). In this case, we considered a mapping scheme between the phenylalanine and the tyrosine in which the hydroxyl group of the latter and the corresponding hydrogen of the former were treated with softcore potentials^49^ (Figure 5C). With this scheme, the aromatic ring—common to both amino acids—preserves the conformation observed in crystal structures, also stably reproducing what is observed in the equilibrium simulation of the *wt* enzyme (Figure S9B). This mapping scheme and sampling resulted in a ΔΔ*G*_b_ estimate of −0.2 ± 0.1 kcal/mol, which perfectly matches the experimental data.

The initial poor agreement of the computed estimates with experimental values for these two mutations, T35S and F28Y, was thus resolved by an *ad hoc* atom mapping scheme. This demonstrates that drastic changes in the original interatomic interactions of the mutated residue with the surroundings can significantly affect the outcome of alchemical free energy calculations. This aspect requires great care when analyzing the MD trajectories of each point mutation.

#### Right Pick of the Initial Conformation for Y32F and V33N

**Figure 6:**
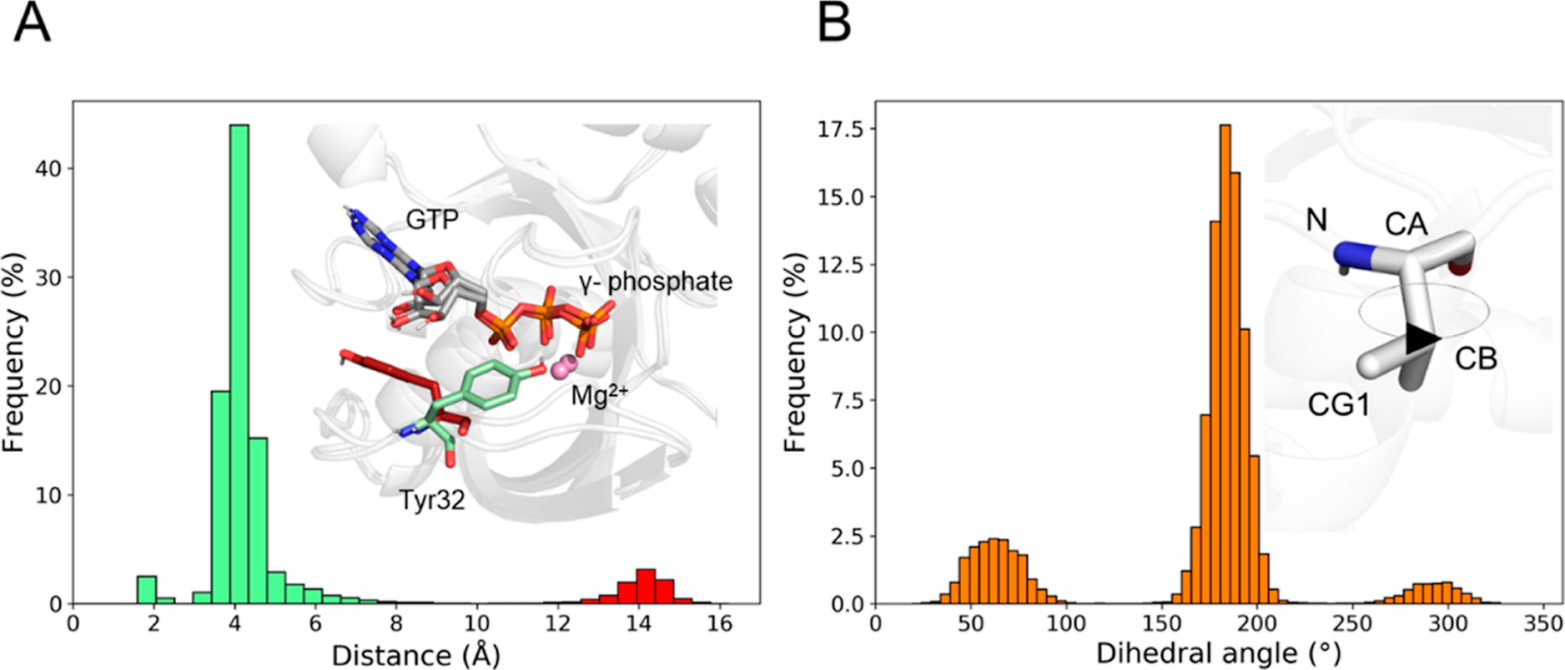
Conformational analysis of Y32 and V33. (A) Distribution of the distance between Tyr32 and the γ-phosphate of GTP. The green and red color codes indicate the most and the least populated Tyr32 conformations, respectively. In the upper right panel, representative conformations of the side chain of Tyr32 observed during the equilibrium MD simulations of the *wt* system are reported. CDC42 is represented as a white cartoon, Tyr32 and the GTP nucleotide in sticks, and the Mg^2+^ ion as a ball. (B) Distribution of the N–CA–CB–CG1 V33 dihedral angle. In the upper right panel, a representative conformation of the side chain of Val33 as observed during the equilibrium MD simulations of the *wt* system is reported. CDC42 is represented as a white cartoon, while Val33 is shown as sticks.

Here, we resolved the apparent poor prediction of these two mutations by looking into the conformational equilibrium of the side chain and how this was sampled in our calculations. In particular, we start showing how the initial structure of Y32F, from which the alchemical transformation starts, can impact the computed ΔΔ*G*_b_. For this mutation, sidechain alchemical transformations returned an estimate of −1.5 ± 0.1 kcal/mol, contrary to the experimental value (0.9 ± 0.3 kcal/mol). We first considered a different mapping, reducing the number of atoms unique to each residue during the alchemical transformation (−OH for the tyrosine and the corresponding −H atom for the phenylalanine). This reduces the error (−0.9 ± 0.04 kcal/mol), although the computed estimate remained negative compared to the positive value from experiments. We thus re-analyzed the MD trajectories of the the *wt* CDC42/PAK1 complex and noted that the configuration used to start the alchemical transformation did not belong to the most populated conformational state of the complex. Indeed, Y32 visits two conformations during the equilibrium MD simulations (Figure 6A). One is predominant over the other (90%, Figure 6A). Repeating the calculation using a structure taken from the most populated conformational state led to a ΔΔ*G*_b_ estimate in line with the experimental determination (0.9 ± 0.1 kcal/mol).

The same issue was observed with the mutation V33N. Sidechain alchemical transformations estimated this mutation to improve binding by −1.3 ± 0.1 kcal/mol, while experimentally it was observed to be neutral (0.20 ± 0.1 kcal/mol). Conformational analysis of the MD trajectories revealed that the configuration used to start the alchemical transformation belonged to a low-populated conformation of V33 (Figure 6B). Indeed, repeating the alchemical transformation starting from a configuration taken from the most populated state of V33 returned a ΔΔG that agreed with the experimental data (−0.1 ± 0.1 kcal/mol).

These two examples show that in real-case scenarios, where the conformational sampling is finite, the initial structure can affect the free energy estimates. A careful analysis of the conformational preference of the mutating residues may thus lead to better free energy estimates.

## Discussion

In this study, we have investigated the use of alchemical binding free energy calculations to compute the change in affinity between two proteins when single-point mutations at the protein–protein interface are inserted. Our test case is the protein complex formed by the small Rho-GTPase CDC42 and its downstream effector PAK1. This test case was chosen for two main reasons: (1) this protein–protein interaction is highly relevant for cancer drug discovery,^25^ and (2) there is a wealth of structural, mutagenesis, and affinity data on such a complex,^27^ which therefore served as a solid benchmark to assess the computed affinity values. In total, we considered 16 single-point mutations. Using experimental and computed data, we obtained a correlation coefficient of 0.91 and a MAE of 0.4 kcal/mol (Figure 3B). Thus, our work demonstrates the predictive power of alchemical binding free energy calculations in the context of protein–protein interactions. This computational procedure can compute how single-point mutations affect such protein–protein complexation. However, such remarkable accuracy could be achieved only through a judicious application of the methodology based on a thorough characterization of the system at hand.

The computed relative binding free energy, ΔΔ*G*_b_, of 12 single-point mutations (out of 16) was in great agreement with the experimental value. Notably, even mutations involving a change of charge in the system (Y32K, D38A, and K135Q), treated with the alchemical co-ion approach, were well reproduced, suggesting that the ion distribution in the simulation box was sufficiently sampled within our protocol.^21,50,51,65^

Conversely, the initial ΔΔ*G*_b_ of four mutations (namely, T35S, F28Y, Y32F, and V33N) was far from the experimental value. These four problematic cases were solved by considering two key factors. The first factor is the chemical nature of the transformation, which defines the alchemical path to transform one residue into another. As exemplified by mutants T35S and F28Y, we could obtain an improved match with the experimental value when we explicitly considered key interactions established by such residues during the transformation. Importantly, we could match the experimental value only when these key interactions were preserved by tuning the atom mapping scheme. In fact, all side-chain atoms were initially considered as unique atoms (and thus treated *via* softcore potentials). In this case, we observed that the side chain of the transformed residue was not able to recover key interactions at the end of the transformation. It is thus advisable, as often remarked in the context of drug design,^47^ to minimize the number of unique atoms. In the case of T35S, a careful definition of the atom mapping scheme allowed us to maintain the interaction of the hydroxyl group with the catalytic Mg^2+^ ion and thus the structural integrity of the site throughout the transformation.

The second factor for improved ΔΔ*G*_b_ concerns the importance of picking the most representative structure of a populated state to start the alchemical transformation from. Ideally, exhaustive sampling would solve this issue as well as the one above. In practice, real-case scenarios may limit the configurational sampling of the chemical structure undergoing alchemical transformation. This was exemplified by the Y32F and V33N mutants. In these cases, we obtained better results when the initial protein structure was representative of the most visited ensemble of configurations retrieved from our equilibrium MD simulations. Thus, a conformational analysis of the system may be propaedeutic to identify the conformational preference of the residue undergoing transformation. This will indicate the best configuration to start the alchemical transformation from, facilitating the proper sampling of significant configurations. This observation is in line with recent studies that report larger errors in binding free energy predictions associated with insufficient sampling or incorrect conformation of the mobile loops.^65−67^ Alternatively, as already implemented in the context of drug design,^68,69^ it may be advisable to perform replicas of the alchemical transformation starting from different configurations retrieved from equilibrium MD. Interestingly, and in line with our results and recommendations, recent studies have reported potential issues in calculating the effect of point mutations in antibodies, in particular, for mutations in which a small residue was turned into a bulky one, suggesting the use of structural prediction methods to identify the most representative structures to start alchemical transformations.^20^

It is worth stressing that both the issues highlighted here—atom mapping and initial conformation of the protein used for the alchemical transformations—are clearly related to the limits of finite sampling during the alchemical transformation (here performed using 12 windows, each simulated for 10 ns). The recommendations we have outlined here are aimed at alleviating the sampling limitations, in analogy to what has been proposed by other authors in the context of drug design.^47^ In our case, we have also found that the MM/GBSA method returned a larger error in the quantification of the effect of each mutation, even when multiple snapshots were considered from MD trajectories of the *wt* and mutated systems. As in other reported studies,^65,70^ in our case, the alchemical free energy calculations outperformed MM/GBSA, with an accuracy that can assist in the design of mutagenesis experiments. The alchemical free energy calculation is thus a powerful method in the context of studying protein–protein interactions too. In this regard, the computer-aided rational design of small bioactive peptides may benefit from the use of this technique by facilitating the identification of high-affinity binders to target proteins with multiple applications in diverse therapeutic areas.^71,72^ Indeed, the use of this method in this context as well as in the context of the design of neutralizing antibodies has recently been explored.^20−23^

Along these lines, more extended protein–protein interfaces, such as the one established between the ACE-2 (receptor angiotensin-converting enzyme II) and the COVID-19 spike proteins,^73^ could be investigated with this approach by trying to unravel the effect of evolutionary mutations on protein–protein binding affinities. We further envisage the use of alchemical binding free energy calculations for the design of variants to characterize signaling pathways and regulatory mechanisms. For example, this approach could be applied to design variants for generating a new active complex or, as an alternative, for inactivating a downstream signal between the protein partners, also in the context of *de novo* protein design.

For these reasons, alchemical free energy calculations to screen mutations would accelerate the identification of those residues that can generate a sizable effect on protein–protein affinity. Thus, our work further corroborates alchemical free energy calculations as a practical computational tool capable of prioritizing those mutants that may lead to larger effects, impacting positively on the efficiency (*i.e.*, economy) of the experimental counterpart. Therefore, this use of alchemical free energy calculations greatly expands its range of applications, extending the current established practice of such calculations in drug discovery to biochemical and mutagenesis studies.

## Conclusions

Alchemical free energy calculations have considerably progressed in recent years thanks to both methodological advances and availability of efficient codes. As a matter of fact, these calculations are routinely employed by academia and industry to guide drug design campaigns with remarkable success.^14,47,61,74^ In this context, we report our results of a benchmark study aimed at assessing the use of alchemical free energy calculations to quantify the effect of point mutations at the protein–protein interface. The question that motivated this study concerned the possibility of using such calculations to design mutagenesis experiments, which are often critical to investigate biochemical pathways and druggable interactions, in which protein–protein contacts have a leading role.

Notably, our test case—the CDC42/PAK1 complex—is highly relevant for cancer drug discovery, being involved in cancer cell invasion and metastasis. Taking advantage of the availability of experimental data, we performed a total of 34 CDC42 alchemical transformations to obtain the computational ΔΔ*G*_b_ of 16 single-point mutations. Although the computed results were in noteworthy agreement with the experimental values, T35S, F28Y, Y32F, and V33N single-point mutations needed an *ad hoc* atom mapping scheme (T35S and F28Y) and a revision of the choice of the initial protein conformation to perform the alchemical free energy transformations (Y32F and V33N). In this way, the comparison with the reported experimental data revealed a correlation coefficient of 0.91 and a MAE of 0.4 kcal/mol (Figure 3B), proving the predictive power of alchemical free energy calculations in the context of protein–protein interactions.

These results are highly encouraging. We have also shown that a careful analysis of the chemical identity and conformational preference of the mutating residue can alleviate sampling issues. Preliminary equilibrium MD simulations of the *wt* system are thus instrumental for a proper setup of alchemical transformations. To conclude, despite the fact that experimental mutagenesis investigations represent a leading practice to study protein–protein interactions, our work shows how alchemical free energy perturbation can be successfully employed to guide the investigation of biochemical pathways, druggable interactions, and *de novo* protein designs.

## Data and Software Availability

PDB files were downloaded from the RCSB Protein Data Bank (https://www.rcsb.org). MODELLER version 10.1 was used for comparative modeling (https://salilab.org/modeller/). AMBER20 was used to perform MD simulations and alchemical free energy calculations (https://ambermd.org/). MM/GBSA calculations were performed using MMPBSA.py version 14.0 included in AmberTools19 (http://ambermd.org/). Error analysis was performed with pyMBAR available at https://github.com/choderalab/pymbar. Force field parameters for GTP and Mg^2+^ were downloaded from http://amber.manchester.ac.uk/. PyMOL(TM) 2.2.3 was used for molecular visualization (https://pymol.org/2/). Coordinate files of model systems are available from the authors upon request.

## Supporting Information Available

Representation of the investigated proteins; structural analysis of available X-ray structures of GTPases/PAK complexes; thermodynamic cycle and alchemical co-ions approach used to compute the relative binding free energies; and analysis of the MD simulations and of the alchemical transformations (PDF)
