# Supplementary material for: Alchemical Free Energy Calculations to Investigate Protein–Protein Interactions: the Case of the CDC42/PAK1 Complex
Source: J Chem Inf Model. 2022 Jun 9;62(12):3023–33. doi: 10.1021/acs.jcim.2c00348 (PMC9241073; doi:10.1021/acs.jcim.2c00348)
Supplement: Supplementary file 1 — ci2c00348_si_001.pdf [file ci2c00348_si_001.pdf]

Supporting Information

Alchemical free energy calculations to investigate protein-protein interactions: the case of the CDC42/PAK1 complex

Maria Antonietta La Serra,[1] Pietro Vidossich,[1] Isabella Acquistapace,[1]

Anand Ganesan,[2,3] Marco De Vivo[1]*

[1]Laboratory of Molecular Modeling and Drug Discovery, Istituto Italiano di Tecnologia, via Morego 30, 16163 Genoa, Italy

[2]Department of Dermatology, University of California, Irvine, CA 92697, USA

[3]Department of Biological Chemistry, University of California, Irvine, CA 92697, USA

* Email: marco.devivo@iit.it

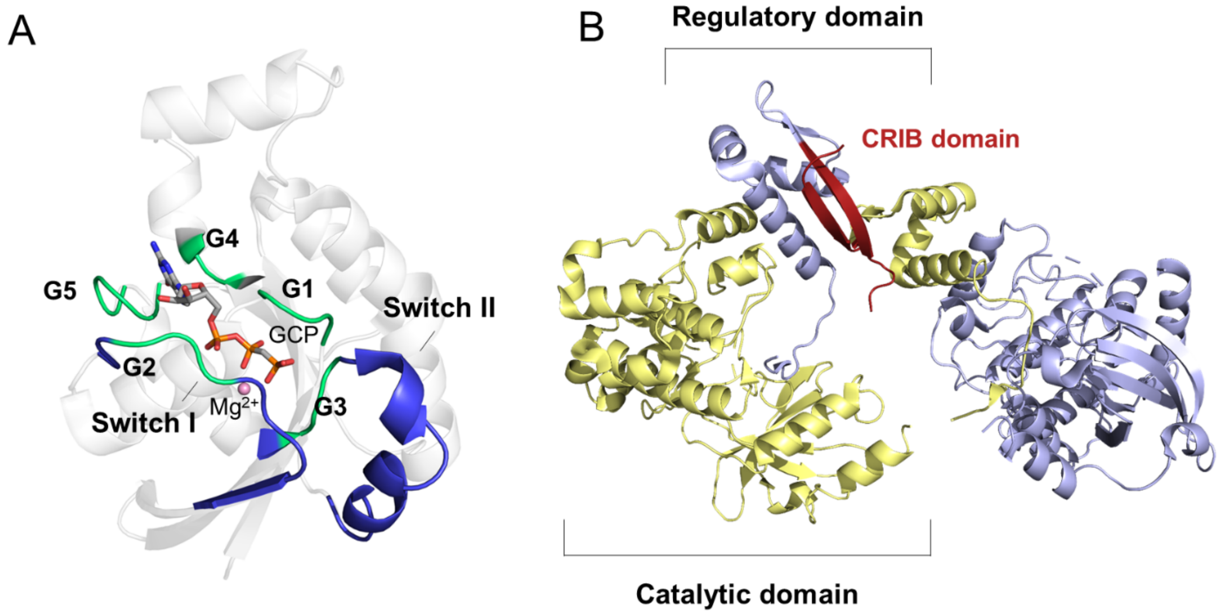

Figure S1. Structural representation of the investigated proteins A) GCP-bound CDC42 (PDB code 2ODB, 2.4 Å resolution). The protein is shown in white cartoon representation, while GCP and Mg^{2+} are shown as sticks and ball, respectively. Conserved motifs around the nucleotide binding site are highlighted in green, while the switch regions are in blue. B) PAK1 (PDB code 1F3M, 2.3 Å resolution). The trans-inhibited dimer is shown in cartoon representation, with individual chains in blue and yellow. The conserved CRIB domain is colored in red in both monomers. Domain labels are used for one monomer.

A

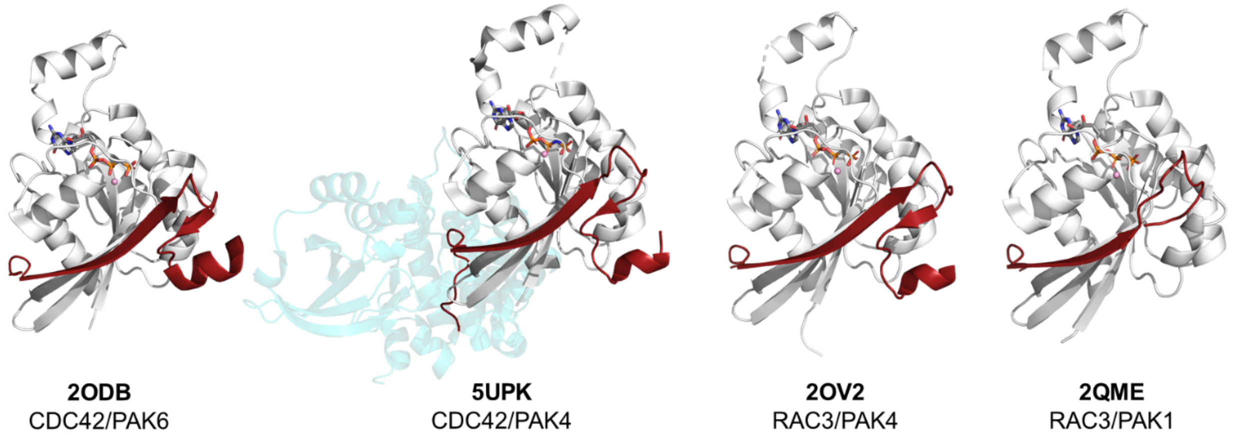

B

	MODEL	2ODB	5UPK	2OV2	2QME
MODEL	0				
2ODB	0.16	0			
5UPK	0.41	0.38	0		
2OV2	0.40	0.34	0.36	0	
2QME	0.42	0.36	0.40	0.30	0

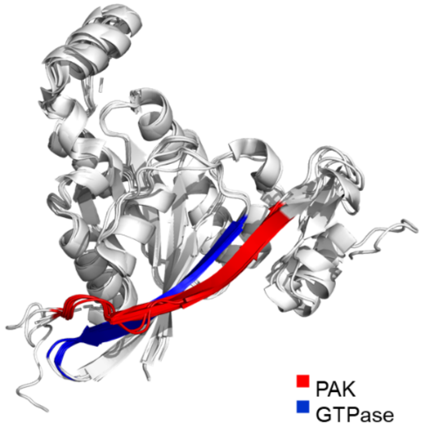

C

	MODEL	2ODB	5UPK	2OV2	2QME
M45-S76	3.0	3.1 (M45-S13)	2.9 (M45-S12)	2.9 (M45-S12)	2.9 (M45-S76)
M45-S76	3.8	3.1 (M45-S13)	2.8 (M45-S12)	3.0 (M45-S12)	2.9 (M45-S76)
T43-S79	2.8	2.8 (T43-Q16)	2.9 (T43-S15)	2.8 (N43-S15)	2.8 (N43-S79)
T43-S79	2.8	2.8 (T43-Q16)	2.7 (T43-S15)	2.8 (N43-S15)	2.9 (N43-S79)
A41-E82	2.9	2.9 (A41-Q19)	3.1 (A41-E18)	2.9 (S41-E18)	2.9 (S41-E82)
A41-E82	2.8	2.8 (A41-Q19)	2.9 (A41-E18)	2.8 (S41-E18)	2.9 (S41-E82)
N39-T84	3.0	2.9 (N39-R21)	3.1 (N39-R20)	2.8 (N39-R20)	2.9 (N39-T84)
N39-I85	3.2	3.2 (N39-V22)	3.4 (N39-V21)	3.3 (N39-V21)	3.2 (N39-I85)
N39-I85	2.9	3.0 (N39-V22)	3.1 (N39-V21)	3.0 (N39-V21)	3.0 (N39-I85)
F37-V87	2.9	3.0 (F37-T24)	2.8 (F37-T23)	2.9 (F37-T23)	2.9 (F37-V87)
G47-E74	5.9	3.6 (G47-E11)	3.0 (G47-E10)	2.8 (D37-E10)	2.8 (D37-E74)

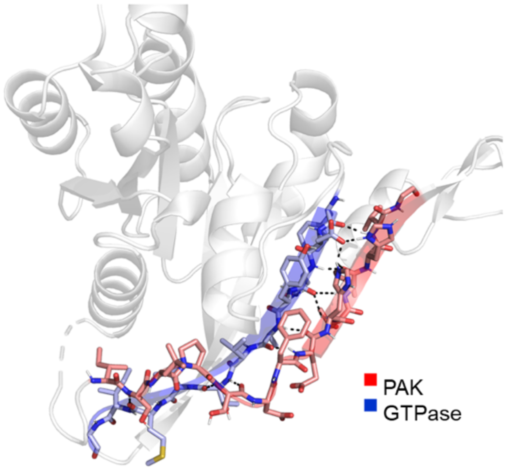

Figure S2. Structural analysis of available X-ray structures of GTPases/PAK complexes. A) The structural representation of the available X-ray structures of GTPases/PAK complexes are reported (from left to right: CDC42/PAK6, PDB code 2ODB, 2.4 Å resolution; CDC/42/PAK4, PDB code 5UPK, 2.4 Å resolution, RAC3/PAK4, PDB code 2OV2, 2.1 Å resolution, RAC3/PAK1, PDB code 2QME, 1.75 Å resolution). GTPase and PAK proteins are represented as white and red cartoon, respectively. B) On the left, the interface RMSD matrix among the described X-ray structures and the CDC42/PAK1 homology model is reported. The RMSD values given in the table are expressed in Å. On the right, the structural representation of the analyzed GTPases/PAK complexes is reported. Interface residues are highlighted as blue and red cartoon for the GTPase and PAK, respectively. C) The distance values of conserved contacts at the β/β-sheets GTPase/PAK interface are reported in the Table. The distance values given in the table are expressed in Å.

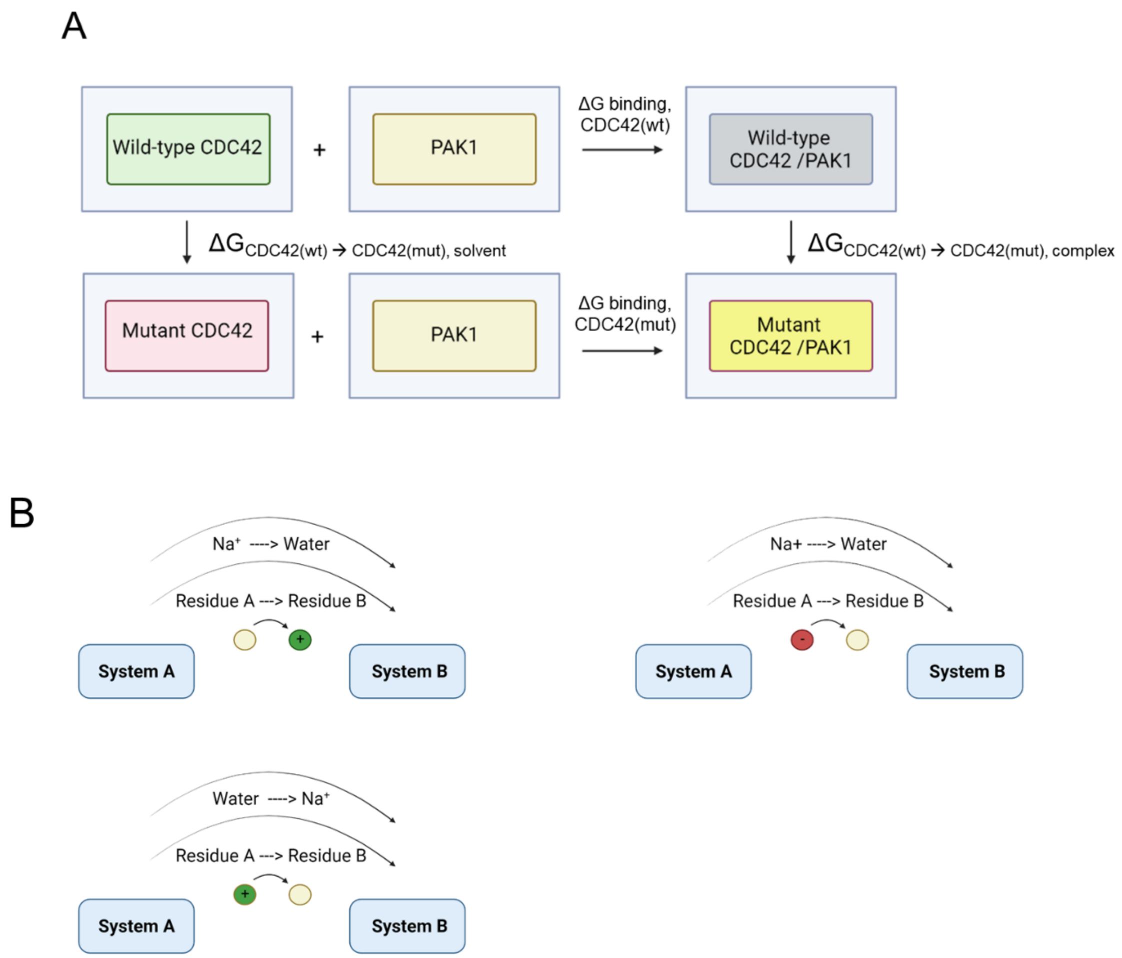

Figure S3. Alchemical binding free energies calculations. A) The thermodynamic cycle used to compute relative binding free energies for the analyzed single-point mutations. Relative binding free energies $\Delta\Delta G_b$ were calculated by subtracting the ΔG_b (wild type CDC42) from the ΔG_b (mutated CDC42). CDC42 was transformed from wild type into mutant in both the apo and PAK1-bound forms. The color code defining the components of the thermodynamic cycle are green for the wild-type CDC42, red for mutant CDC42, beige for PAK1, grey for the wild-type CDC42/PAK1 complex and, finally, yellow for the mutant CDC42/PAK1 complex. B) The scheme represents the alchemical co-ions approach used to treat mutations which involve residues of different charge. For the single-point mutations implying a change in net charge of the protein, either a Na^+ counterion was converted into a water molecule or vice versa. Specifically, for the Y32K mutation the positive charge acquired by the protein was counterbalanced by the concomitant transformation of a Na^+ ion into a water molecule; for the D38A mutation the disappearing negative charge on the protein was proceeded with the concomitant transformation of a Na+ ion into a water molecule; and, lastly, for the K135Q mutation the disappearing positive charge on the protein was compensated by the concomitant transformation of a water molecule in a Na^+ ion.

A

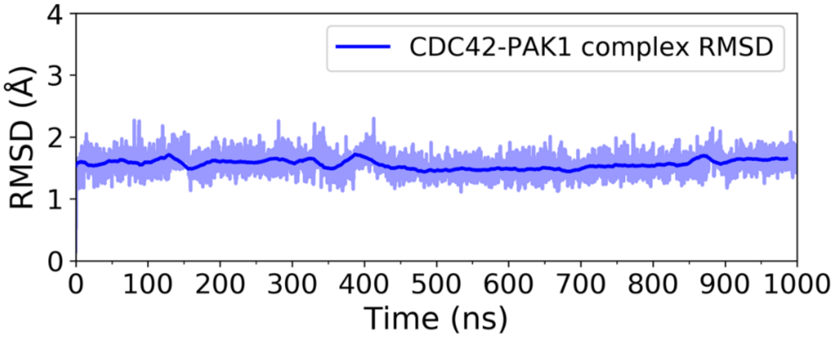

CDC42-PAK1 complex RMSD
RMSD (Å)
Time (ns)

B

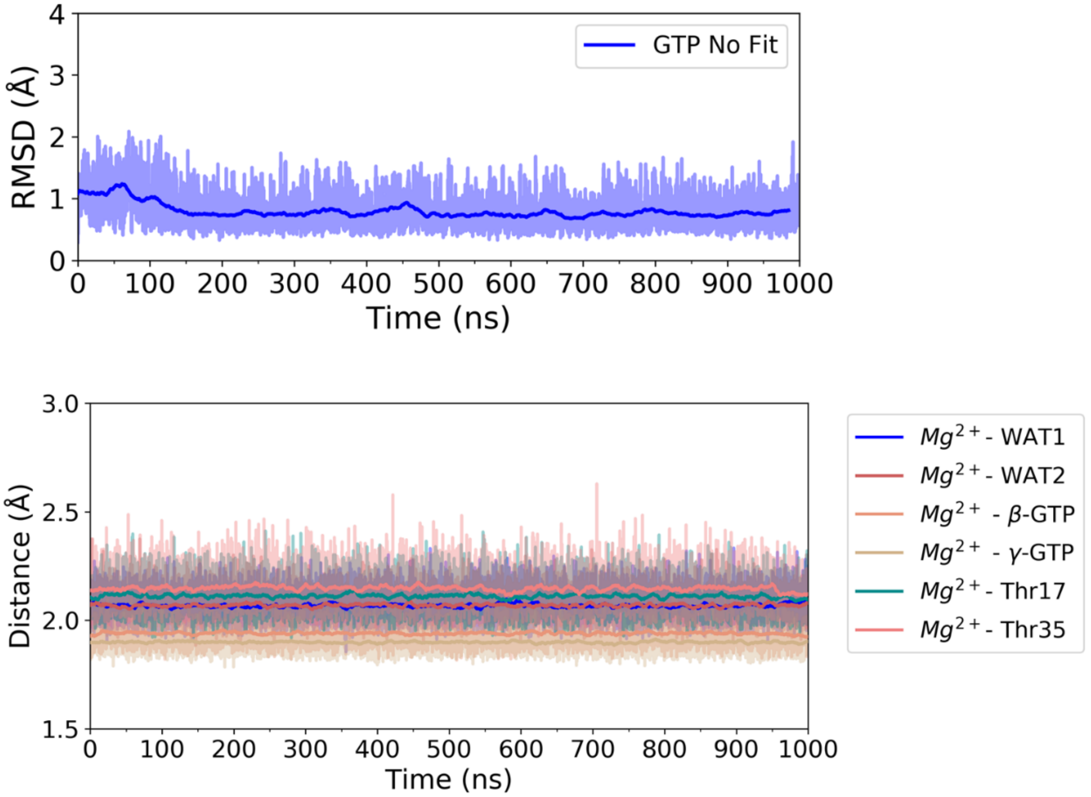

GTP No Fit
RMSD (Å)
Time (ns)
Distance (Å)
Mg^{2+} - WAT1
Mg^{2+} - WAT2
Mg^{2+} - β-GTP
Mg^{2+} - γ-GTP
Mg^{2+} - Thr17
Mg^{2+} - Thr35
Time (ns)

C

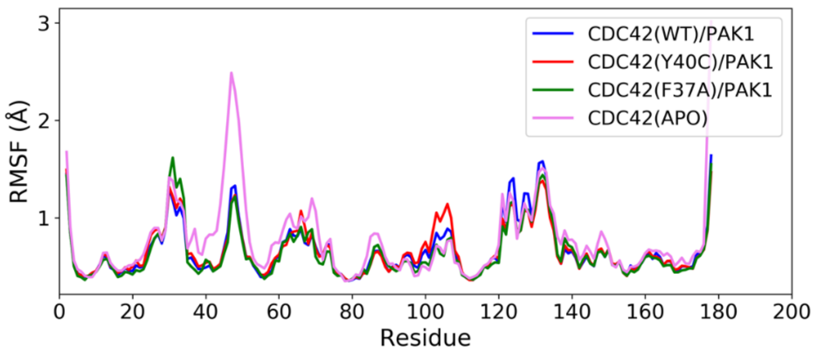

Figure S4. MD simulations of CDC42/PAK1 complexes. A) The time series RMSD descriptor for the system's backbone of CDC42(wt)/PAK1 is reported. B) The conservation of the GTP catalytic pocket was investigated through the time series RMSD for the GTP nucleotide (B, on the top) and by monitoring the length of the Mg^{2+} coordination bonds. Both RMSD and distance values descriptors are shown as shaded areas, while the associated running averages are represented as lines. C) The Root mean square fluctuation (RMSF) per residue of CDC42 alone and in CDC42/PAK1 complexes is reported. Compared to the unbound CDC42, in all the case studies the formation of the complex reduces the fluctuations of residues located at the interface.

A

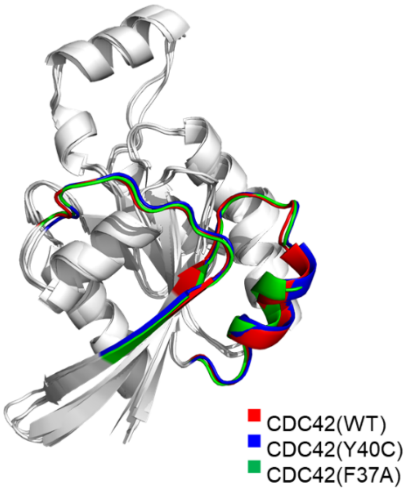

	CLUSTER$_{WT}$	CLUSTER$_{Y40C}$	CLUSTER$_{F37A}$
CLUSTER$_{WIT}$	0		
CLUSTER$_{Y40C}$	0.48	0	
CLUSTER$_{F37A}$	0.84	0.84	0

B

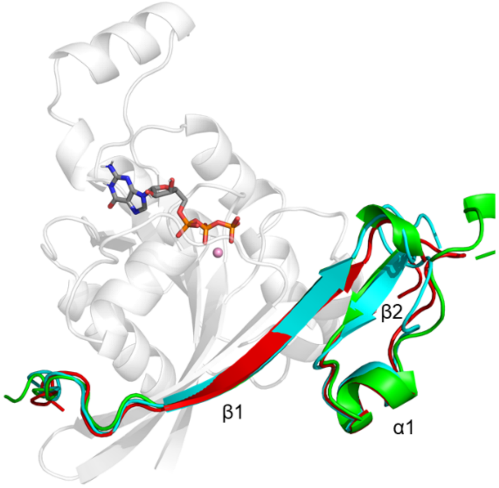

Full length PAK1

	CLUSTER$_{WT}$	CLUSTER$_{Y40C}$	CLUSTER$_{F37A}$
CLUSTER$_{WT}$	0		
CLUSTER$_{Y40C}$	0.80	0	
CLUSTER$_{F37A}$	0.76	0.71	0

β1 PAK1

	CLUSTER$_{WT}$	CLUSTER$_{Y40C}$	CLUSTER$_{F37A}$
CLUSTER$_{WT}$	0		
CLUSTER$_{Y40C}$	0.73	0	
CLUSTER$_{F37A}$	1.12	1.33	0

β2 PAK1

	CLUSTER$_{WT}$	CLUSTER$_{Y40C}$	CLUSTER$_{F37A}$
CLUSTER$_{WT}$	0		
CLUSTER$_{Y40C}$	1.44	0	
CLUSTER$_{F37A}$	1.24	0.88	0

α1 PAK1

	CLUSTER$_{WT}$	CLUSTER$_{Y40C}$	CLUSTER$_{F37A}$
CLUSTER$_{WT}$	0		
CLUSTER$_{Y40C}$	1.77	0	
CLUSTER$_{F37A}$	1.49	1.33	0

Figure S5. Switch motifs and PAK1 clustering analysis. A) On the left, superposition of representative structures of the clusters obtained by grouping the configurations along the MD trajectories of CDC42/PAK1 complexes based on the RMSD of the switch regions of CDC42. Switches residues are highlighted as red, blue and green cartoon for the wild-type, Y40C and F37A CDC42 forms, respectively. On the right, RMSD values of the switch regions expressed in Å. B) On the left, superposition of representative structures of the clusters obtained by grouping the configurations along the MD trajectories of CDC42/PAK1 complexes based on the RMSD of PAK1. PAK1 are highlighted as green, red and cyan cartoon for the wild-type, Y40C and F37A CDC42 forms, respectively. On the right, RMSD values of different structural elements of PAK1 (expressed in Å).

A

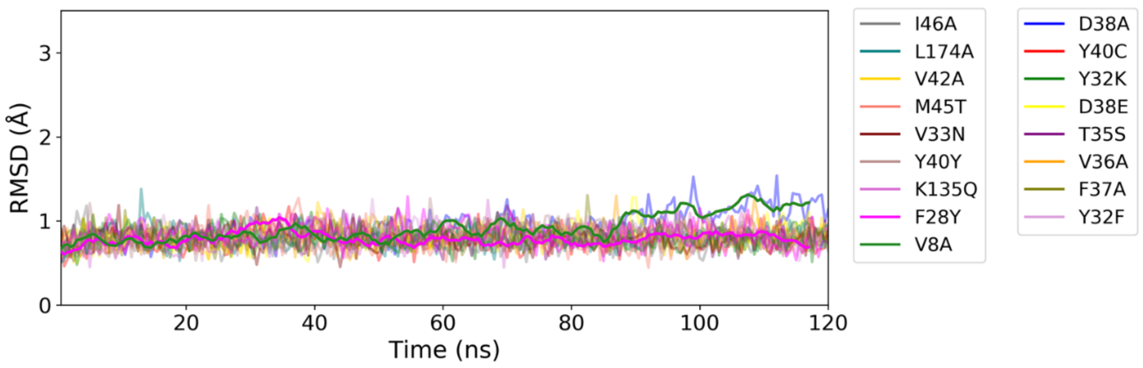

Figure S6. CDC42/PAK1 alchemical simulations. The time series of the RMSD of the backbone of interface residues is reported for each investigated mutation

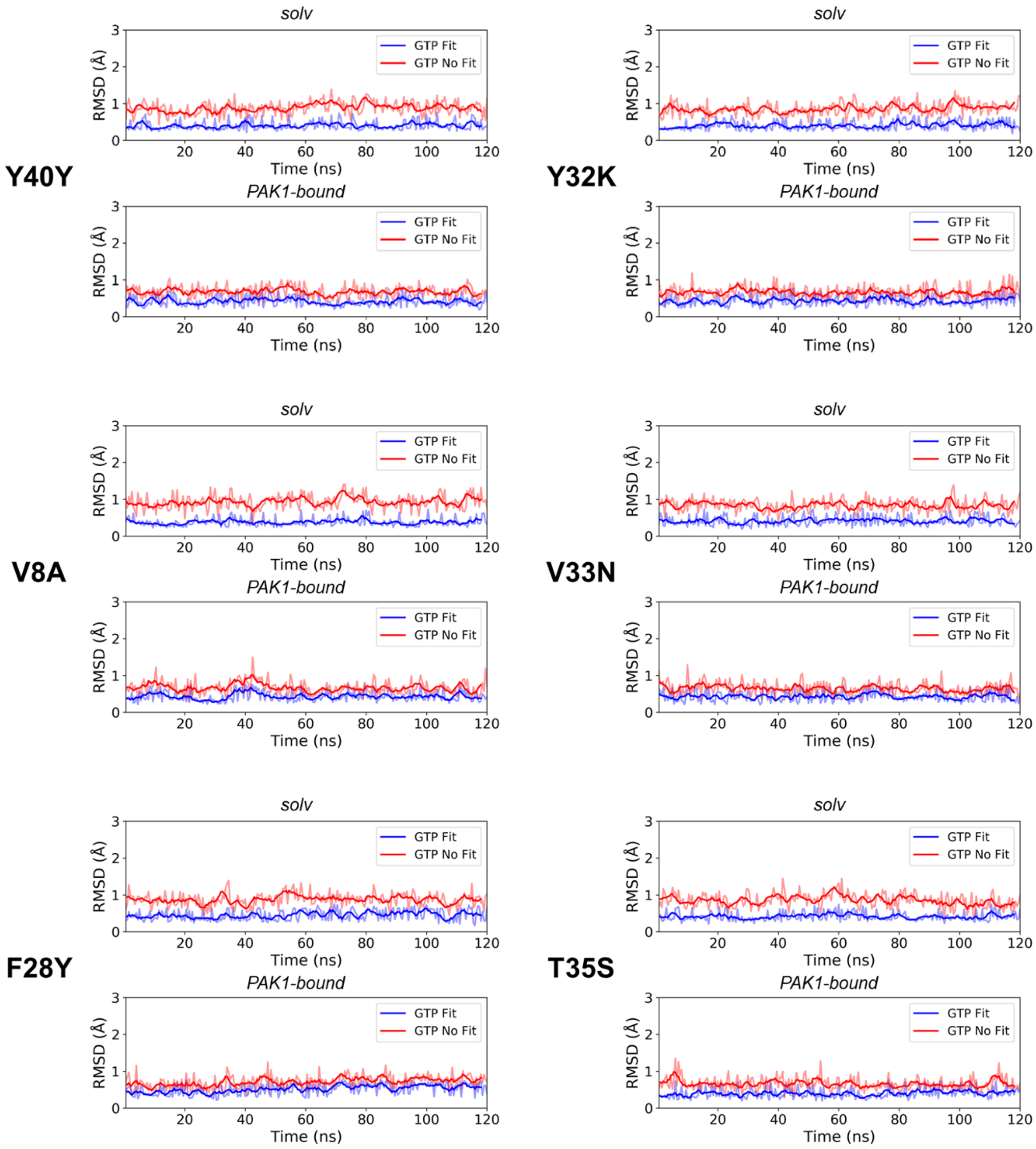
Y40Y
Y32K
V8A
V33N
F28Y
T35S
solv
PAK1-bound
GTP Fit
GTP No Fit
RMSD (Å)
Time (ns)

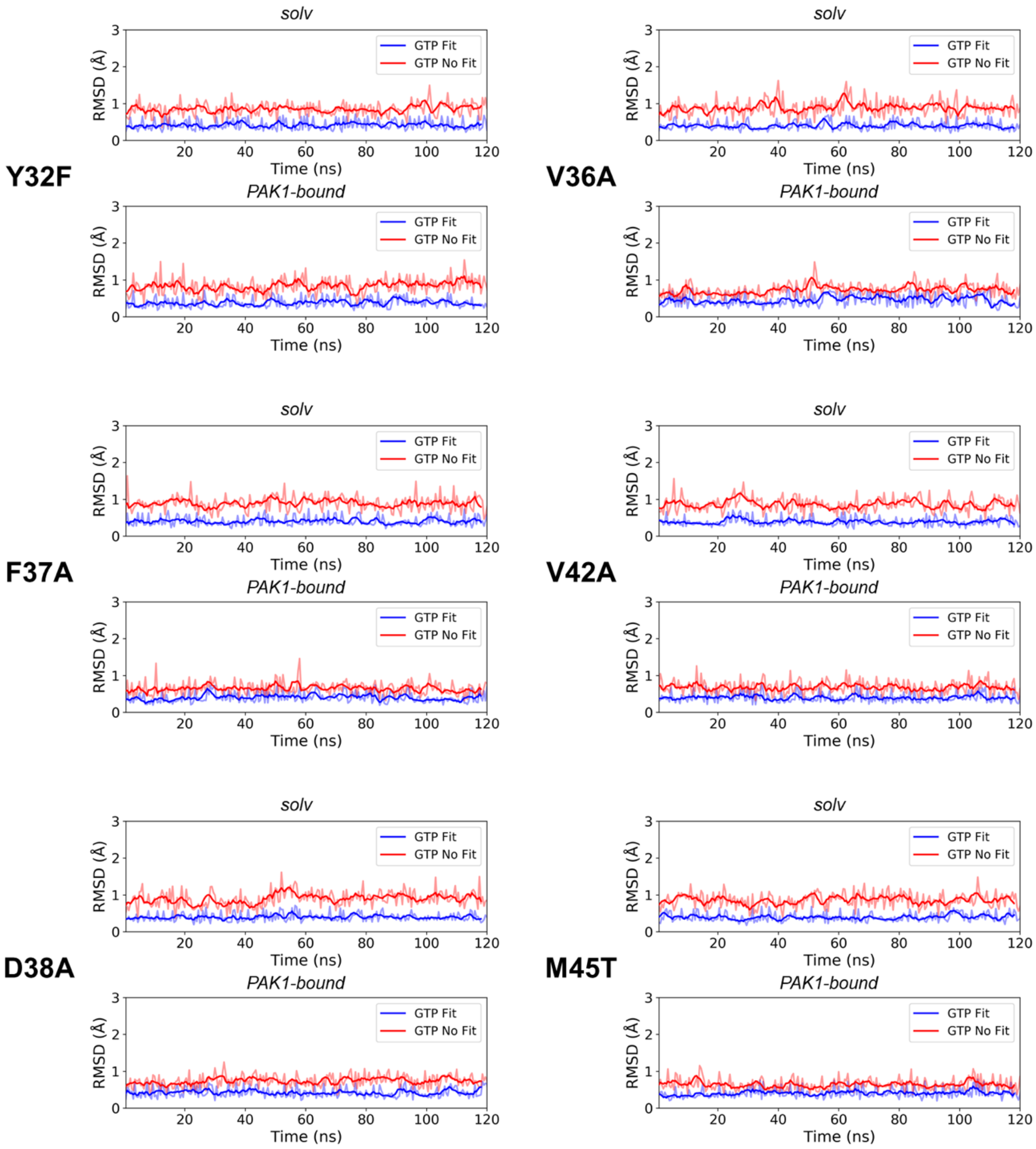
Y32F
V36A
F37A
V42A
D38A
M45T
solv
PAK1-bound
GTP Fit
GTP No Fit
RMSD (Å)
Time (ns)

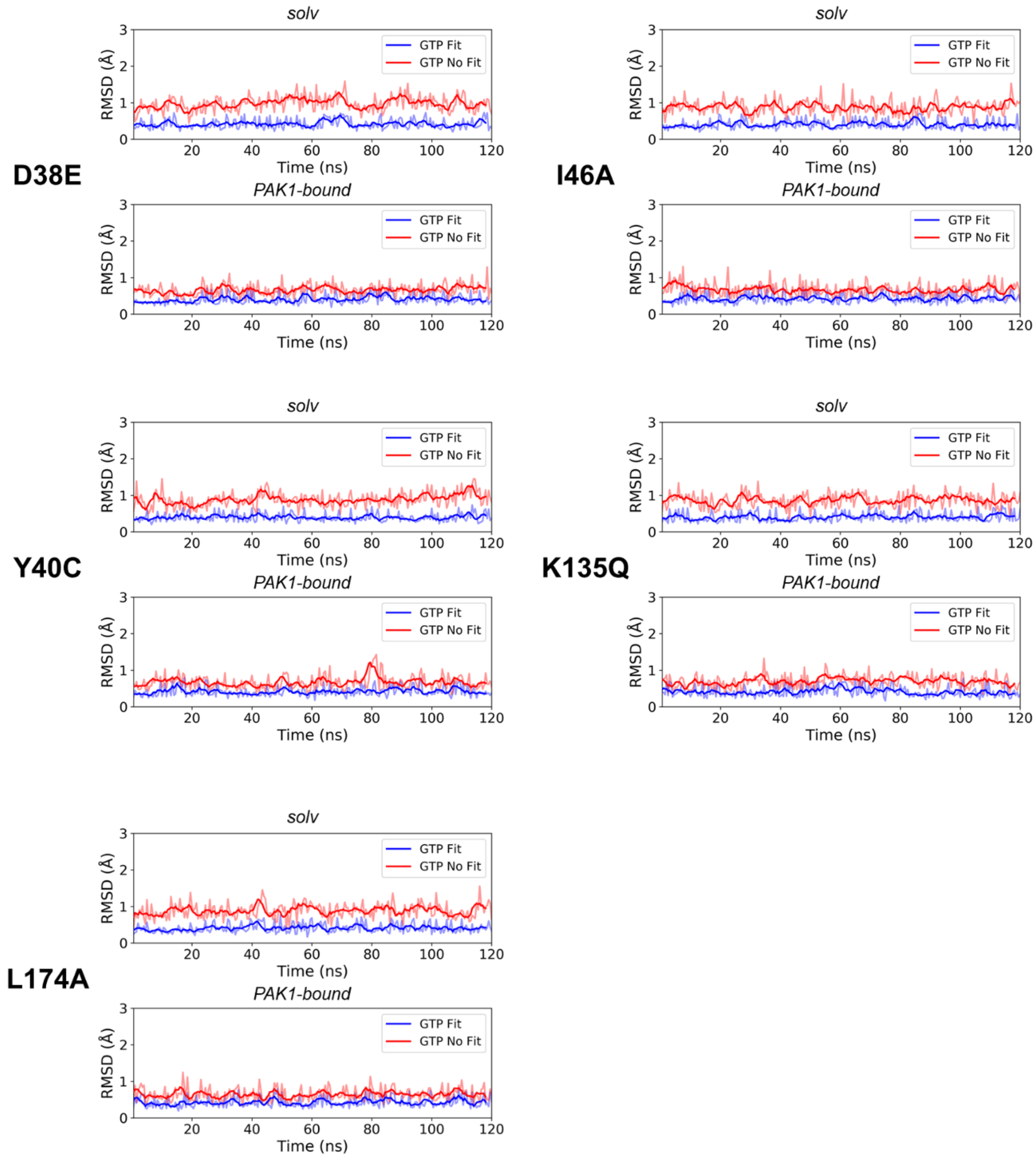

Figure S7. CDC42/PAK1 and CDC42 alchemical simulations. For each investigated mutation, the time series of the RMSD of the GTP nucleotide for both apo and PAK1-bound CDC42 forms are reported. The RMSD was computed after aligning the nucleotide (blue line) and after aligning the protein backbone (red line).

A

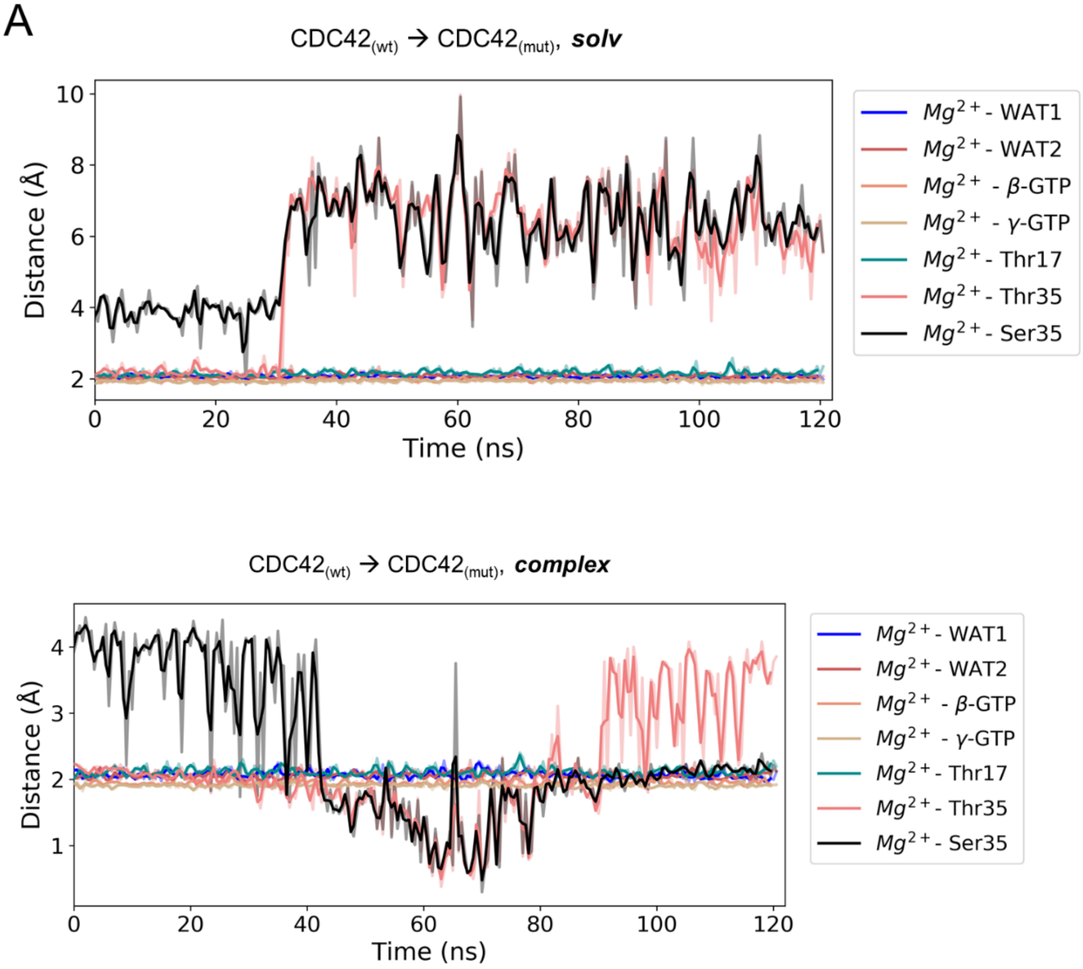

CDC42(wt) → CDC42(mut), solv
Distance (Å)
Time (ns)
Mg2+ - WAT1
Mg2+ - WAT2
Mg2+ - β-GTP
Mg2+ - γ-GTP
Mg2+ - Thr17
Mg2+ - Thr35
Mg2+ - Ser35
CDC42(wt) → CDC42(mut), complex
Distance (Å)
Time (ns)
Mg2+ - WAT1
Mg2+ - WAT2
Mg2+ - β-GTP
Mg2+ - γ-GTP
Mg2+ - Thr17
Mg2+ - Thr35
Mg2+ - Ser35

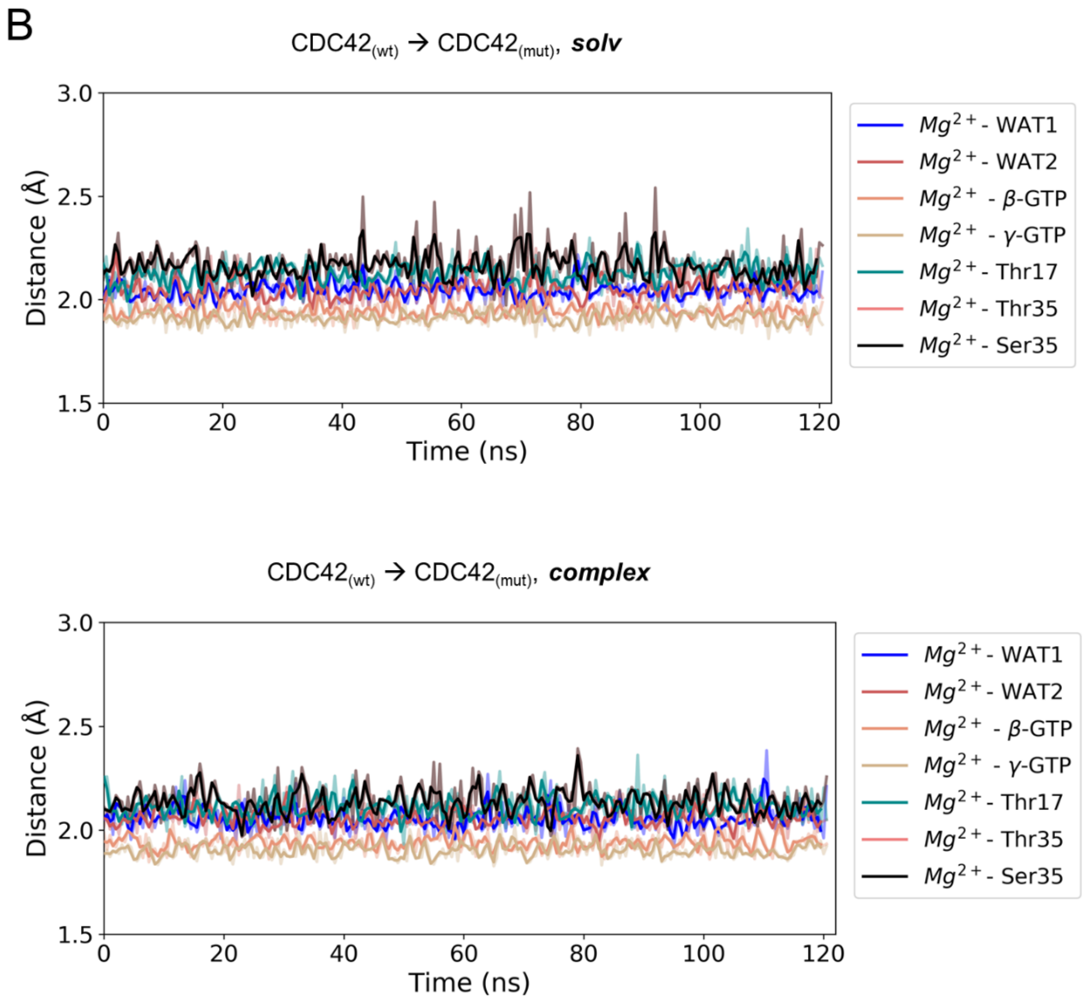

Figure S8. Alchemical simulations of the T35S mutation. The time series of the Mg^{2+} coordination bonds lengths for both unbound and PAK1-bound CDC42 are reported for two atom mapping schemes: A) whole side chains transformation and B) minimal softcore atoms (see text).

A

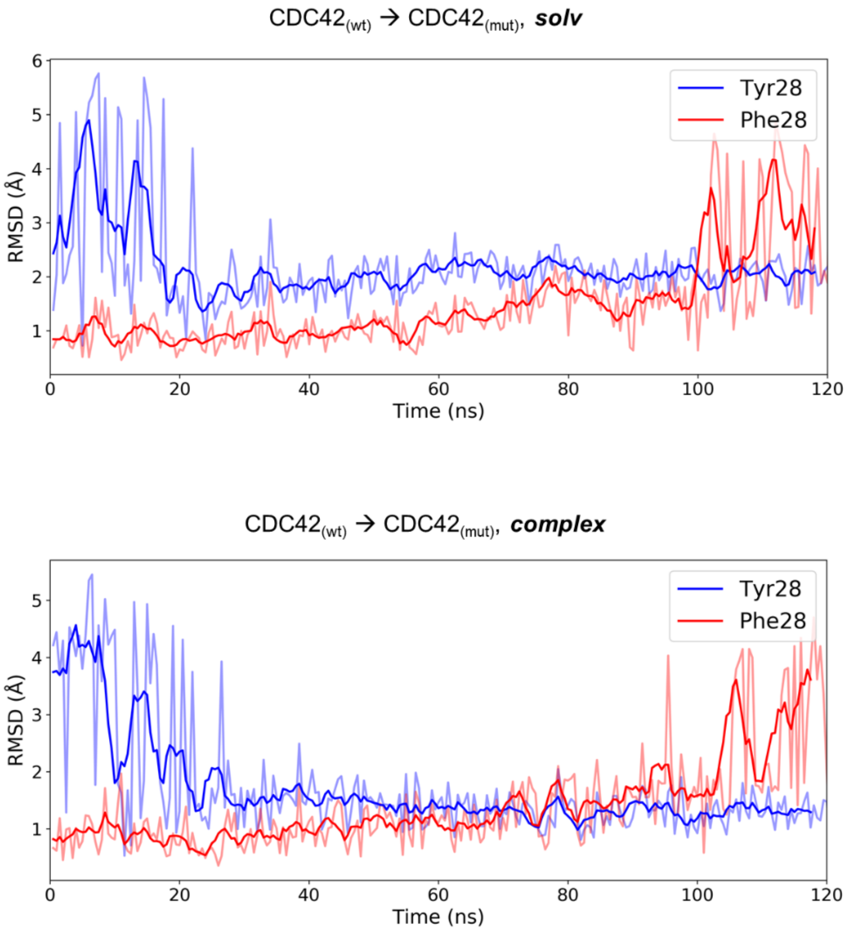
CDC42$_{(wt)}$ → CDC42$_{(mut)}$, ***solv***
Tyr28
Phe28
RMSD (Å)
Time (ns)
CDC42$_{(wt)}$ → CDC42$_{(mut)}$, ***complex***
Tyr28
Phe28
RMSD (Å)
Time (ns)

B

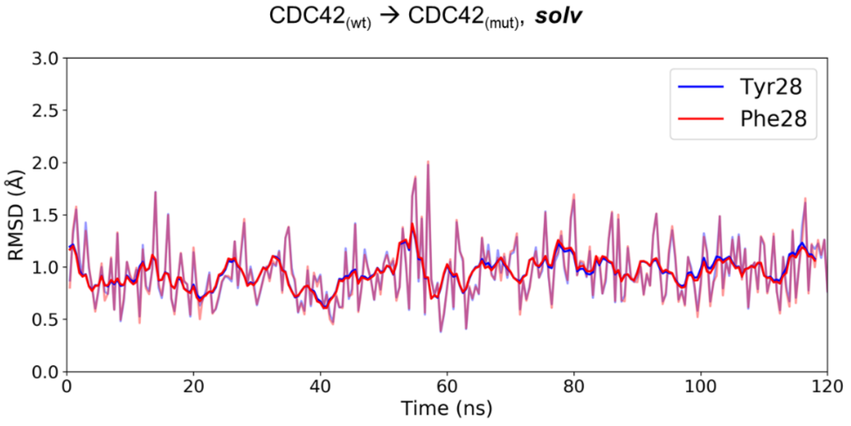
CDC42$_{(wt)}$ → CDC42$_{(mut)}$, ***solv***
Tyr28
Phe28
RMSD (Å)
Time (ns)

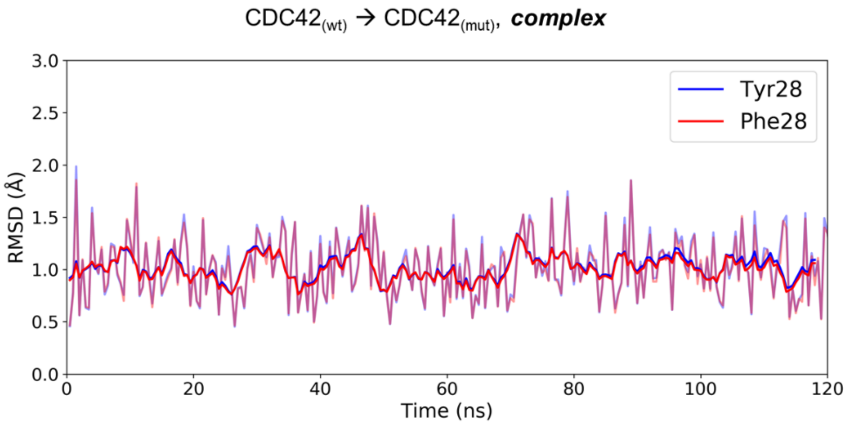

Figure S9. Alchemical simulations of the F28Y mutation. The time series of the RMSD of residues F28 and Y28 for both unbound and PAK1-bound CDC42 are reported for two atom mapping schemes: A) whole side chains transformation and B) minimal softcore atoms (see text).

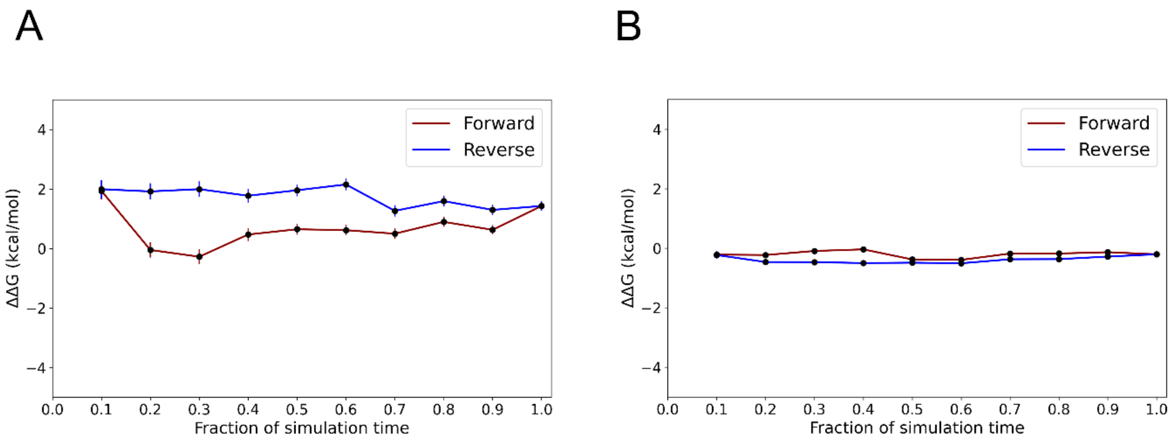

Figure S10. Convergence analysis of the computed $\Delta\Delta G_b$ for the F28Y alchemical simulations. The convergence of the computed ΔΔGb values was assessed estimating it as a function of simulation time, considering intervals both in the forward and the reverse direction as proposed by Klimovich *et al.* (*J Comput Aided Mol Des* 2015, 29, 397). A) Convergence of $\Delta\Delta G_b^{comp}$ based on sidechain alchemical transformations and B) after tuning the atom mapping scheme. The improved convergence properties observed in panel B highlight a further advantage of the *ad-hoc* atom mapping scheme.
